# Triangular Topological 2D Covalent Organic Frameworks Constructed via Symmetric or Asymmetric “Two‐in‐One” Type Monomers

**DOI:** 10.1002/advs.202105517

**Published:** 2022-02-10

**Authors:** Weiben Chen, Pei Chen, Dan Chen, Yi Liu, Guang Zhang, Lei Wang, Long Chen

**Affiliations:** ^1^ Shenzhen Key Laboratory of Polymer Science and Technology Guangdong Research Center for Interfacial Engineering of Functional Materials College of Materials Science and Engineering Shenzhen University Shenzhen 518060 China; ^2^ College of Physics and Optoelectronic Engineering Shenzhen University Shenzhen 518060 China; ^3^ Department of Chemistry Tianjin Key Laboratory of Molecular Optoelectronic Science Tianjin University Tianjin 300072 China; ^4^ State Key Laboratory of Supramolecular Structure and Materials College of Chemistry Jilin University Changchun 130012 China

**Keywords:** asymmetric, covalent organic frameworks, hexaphenylbenzene, triangular topology

## Abstract

Most of the reported covalent organic frameworks (COFs) so far are prepared from highly symmetric building blocks, which to some extent limits the expansion of COF diversity and complexity. Low‐symmetric building blocks can be designed through a desymmetrized vertex strategy, which might be used to construct new topological COFs. But reports of COFs constructed by asymmetric building blocks are thus far very rare. Here, a feasible strategy to design asymmetric building blocks for COF synthesis is introduced, by simply varying the positions of functional groups in the monomer. As a proof of concept, two isomeric hexaphenylbenzene‐based “two‐in‐one” type monomers (1,2,4‐HPB‐NH_2_ and 1,3,5‐HPB‐NH_2_) are designed and synthesized. To the authors’ surprise, self‐polycondensation of the asymmetric 1,2,4‐HPB‐NH_2_ (i.e., the isomer of common *C_3_*‐symmetric 1,3,5‐HPB‐NH_2_) also affords highly crystalline COF (1,2,4‐HPB‐COF) similar to the symmetric 1,3,5‐HPB‐NH_2_ counterpart with identical topological structure. The triangular porous structures of both HPB‐based COFs are well resolved by powder X‐ray diffraction (PXRD), theoretical simulations, nitrogen sorption, and morphologies analysis. This work demonstrates the “two‐in‐one” type asymmetric building blocks can also produce highly crystalline frameworks and thus provides a new structural design strategy for reticular chemistry.

## Graphical abstract

A branch‐exchange desymmetrization molecular design strategy is demonstrated by synthesizing two isomeric hexaphenylbenzene‐based “two‐in‐one” type monomers (1,2,4‐HPB‐NH_2_ and 1,3,5‐HPB‐NH_2_). Self‐polycondensation of the asymmetric monomer 1,2,4‐HPB‐NH_2_ can also form highly crystalline triangular topological 1,2,4‐HPB‐COF, which is almost the same as that of 1,3,5‐HPB‐COF prepared by the *C*_3_‐symmetric 1,3,5‐HPB‐NH_2_ isomer. The structures and electronic properties of these two HPB‐COFs are systematically investigated.

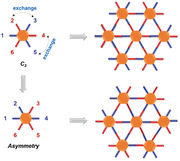

## 1 Introduction

**Scheme 1:**
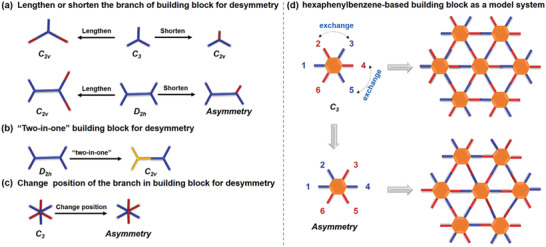
a–c) representative design tactics for lowering the symmetry of building blocks; d) a strategy to decrease the symmetry of the “two‐in‐one” hexaphenylbenzene‐based building block by changing the positions of the branches.

Covalent organic frameworks (COFs) as a new class of crystalline and porous organic materials^[^ ^1^ ^]^ have received tremendous research interests in versatile fields, e.g. gas storage and separation,^[^ ^2^ ^]^ catalysis,^[^ ^3^ ^]^ water splitting,^[^ ^4^ ^]^ fluorescence sensing,^[^ ^5^ ^]^ batteries,^[^ ^6^ ^]^ environmental remediation,^[^ ^7^ ^]^ and photoelectric devices.^[^ ^8^ ^]^ Their promising application potentials benefit from the intriguing structural features, including highly ordered crystalline skeletons, large and permanent porosities, high stability, and tunable functionality and topologies.^[^ ^9^ ^]^ Since the first report by Yaghi et al.,^[^ ^10^ ^]^ tremendous synthetic efforts have been devoted toward high‐quality COFs with fascinating structures and functions.^[^ ^11^ ^]^ Choosing suitable building blocks is crucial to precisely regulate and obtain the targeted COFs with desired topological structures. Up to now, the highly symmetric building blocks are most commonly used for the construction of COFs. For example, the hexagonal or quadrangular COFs can be readily prepared using *C_2_‐*symmetric linear linkers with *C_3_* or *C_4_‐*symmetric vertices.^[^ ^12^ ^]^ However, reports on trigonal porous COFs^[^ ^13^ ^]^ through reacting *C_6_*‐ and *C_2_*‐symmetric building blocks are much sparse due to the limitation of suitable building units, while the triangular topology usually exhibits smaller aperture and higher *π*‐column density than those of 2D hexagonal and tetragonal structures as reported by Jiang et al.^[^ ^13a^ ^]^ Low‐symmetric building blocks can also be utilized to construct diverse COFs.^[^ ^14^ ^]^ For example, Zhang et al. exploited a desymmetrized vertex design approach through lengthening or shortening the branches of high‐symmetry building blocks (e.g., *C_3_*/*D_3h_* or *D_2h_*, **Scheme** **1**a) to afford low‐symmetry (*C_2v_*) or even asymmetric ones, which can form a variety of heteroporous COFs (Scheme 1a).^[^ ^15^ ^]^ Recently, we reported a “two‐in‐one” molecular design strategy to lower the symmetry of the building blocks by integrating bifunctional groups (e.g., aldehyde and amino groups) in the same monomer (Scheme 1b).^[^ ^16^ ^]^ Besides the above‐mentioned strategies for desymmetrization, changing positions of the branches is another method to lower the symmetry but has not been reported, probably owing to the lack of applicable building blocks as a model system.

Fortunately, hexaphenylbenzene (HPB)‐based building blocks can be used as an ideal example to demonstrate the third desymmetrization approach by reversing the positions of the four branches. The six peripheral phenyl substitutes in HPB were arranged in the central benzene core with nearly identical angles between two peripheral branches (60°).^[^ ^17^ ^]^ The six external benzene rings and the almost fixed angle favor altering the position of the branch to regulate the symmetry. In addition, several synthetic routes to prepare HPB derivatives are available, such as Diels−Alder reaction, cyclotrimerization of diphenylacetylenes, and Suzuki–Miyaura coupling reaction.^[^ ^18^ ^]^ Among them, cyclotrimerization of asymmetric functionalized diphenylacetylenes can form two isomers, i.e., the *C_3_‐*symmetric 1,3,5‐substituted isomer and the asymmetric 1,2,4‐substituted counterpart. The asymmetric 1,2,4‐substituted isomer could be considered as position exchange between sites 3 and 5 and between sites 2 and 4 of the highly symmetric 1,3,5‐substituted isomer, which meets the aforementioned desymmetry strategy by altering the positions of the branches (Scheme 1d). Owing to the dynamic nature of imine formation and error‐correction process in COF formation, we assume that the asymmetric 1,2,4‐substituted isomer, a new “two‐in‐one” type building block with equivalent reaction groups, can also potentially form triangular topological COFs similar to that obtained from the 1,3,5‐substituted building blocks (Scheme 1d).

**Figure 1:**
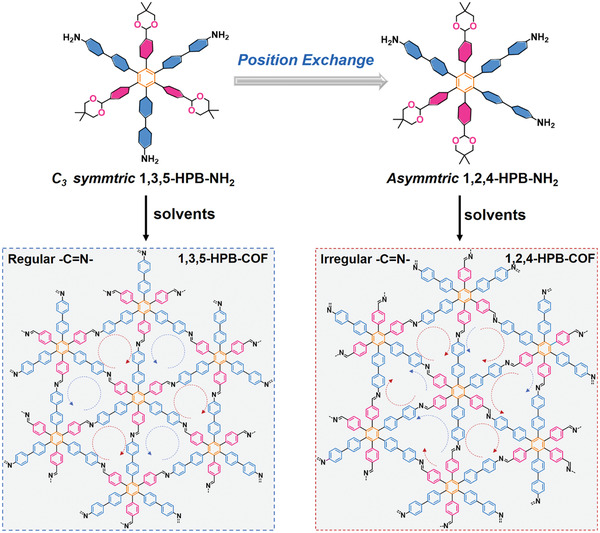
Synthetic routes of 1,3,5‐HPB‐COF from *C_3_‐*symmetric 1,3,5‐HPB‐NH_2_ and 1,2,4‐HPB‐COF from asymmetric 1,2,4‐HPB‐NH_2_. The —C═N— linkages are regularly and irregularly arranged in the framework of 1,3,5‐HPB‐COF and 1,2,4‐HPB‐COF, respectively as highlighted by the dashed arrows.

To prove this hypothesis, we designed and synthesized two model building blocks, i.e., 1,3,5‐HPB‐NH_2_ and 1,2,4‐HPB‐NH_2_ (**Figure** **1**). The formyl groups were protected with neopentyl glycol to avoid possible partial self‐polycondensation of the bifunctional monomers during the synthesis and work‐up process.^[^ ^16d^ ^]^ We subsequently screened different solvent systems and concentrations of two isomers to prepare the corresponding COFs. Thanks to the solvent adaptability of the “two‐in‐one” strategy, both asymmetric 1,2,4‐HPB‐NH_2_ and *C_3_‐*symmetric 1,3,5‐HPB‐NH_2_ in specific simplex solvents can produce highly crystalline networks, i.e., 1,2,4‐HPB‐COF and 1,3,5‐HPB‐COF respectively, as verified by the powder X‐ray diffractions (PXRD) results. Interestingly, the PXRD profiles of 1,2,4‐HPB‐COF are nearly the same as those of 1,3,5‐HPB‐COF. Because of the nonsymmetry of 1,2,4‐HPB‐NH_2_ and disorder of —C═N— in framework, it is too difficult to distinguish different possibilities and directly simulate the long‐range ordering structure of triangular 1,2,4‐HPB‐COF on Materials Studio software (Figure 1; Figure S1, Supporting Information).^[^ ^19^ ^]^ We first simulated PXRD of the triangular topological 1,3,5‐HPB‐COF, which matched well with the experimental PXRD patterns of 1,2,4‐HPB‐COF. Experimental pore sizes of 1,2,4‐HPB‐COF were in line with the simulated triangular apertures. Additionally, both 1,3,5‐HPB‐COF and 1,2,4‐HPB‐COF synthesized from the same solvent exhibit similar morphologies. Our work demonstrates that asymmetric monomers could be applied to construct 2D COFs as well, which not only increases the diversity and complexity of 2D COF library, but also provides an alternative pathway to engineer the structures and topologies of 2D COFs.

## 2 Results and Discussion

To synthesize the two targeted isomeric monomers (1,3,5‐HPB‐NH_2_ and 1,2,4‐HPB‐NH_2_), the isomeric intermediates (1,3,5‐HPB‐Br and 1,2,4‐HPB‐Br) were first prepared by cobalt‐catalyzed cyclotrimerization of the asymmetric tolan precursor (Scheme S1, Supporting Information). Fortunately, the isomeric 1,3,5‐HPB‐Br and 1,2,4‐HPB‐Br were successfully separated by common silica gel column chromatography owing to their different polarities. Furthermore, 1,3,5‐HPB‐NH_2_ and 1,2,4‐HPB‐NH_2_ were synthesized by the Sukuzi–Miyaura coupling reaction of 4‐(4,4,5,5‐tetramethyl‐1,3,2‐dioxaborolan‐2‐yl)aniline with 1,3,5‐HPB‐Br and 1,2,4‐HPB‐Br, respectively (Schemes S2 and S3, Supporting Information). Nuclear magnetic resonance (NMR) and high‐resolution mass spectra further demonstrated the successful preparation of the two targeted isomers (see Supporting Information). Similar to our previous reported COFs via “two‐in‐one” molecular design strategy, the symmetric 1,3,5‐HPB‐NH_2_ can facilely afford highly crystalline networks (denoted as 1,3,5‐HPB‐COF_solvents_) in various simplex solvents (Figure S3, Supporting Information). To demonstrate the asymmetric 1,2,4‐isomer can also be used to synthesize crystalline COFs, we first screened different solvents and different amounts of acetic acid at 120 °C for 3 days under solvothermal conditions (see Supporting Information). As shown in Figures S2 and S3 (Supporting Information), 1,2,4‐HPB‐NH_2_ can also afford crystalline powders (denoted as 1,2,4‐HPB‐COF_solvents_). Better crystallinity can be achieved in five different alcohols as the reaction solvents using 300 µL of 6 m acetic acid as the condensation catalyst.

Both 1,2,4‐HPB‐COF and 1,3,5‐HPB‐COF were unambiguously characterized by a variety of analytical techniques. As revealed in the Fourier transform infrared (FT‐IR) spectra (Figure S4, Supporting Information), the alkyl bands ranging 2900–3000 cm^–1^ and the N‐H stretching vibrations of amino groups at ≈3300 cm^–1^ in 1,2,4‐HPB‐NH_2_ monomer were significantly attenuated in the IR spectra of 1,2,4‐HPB‐COF. Furthermore, a new band appeared ≈1626 cm^–1^ for 1,2,4‐HPB‐COF that was attributed to the stretching vibrations of —C═N— linkages. Similar results were observed when comparing the IR spectra of 1,3,5‐HPB‐COF with that of 1,3,5‐HPB‐NH_2_. These results suggest a high polymerization degree for both HPB‐COFs. Solid‐state cross‐polarization magic‐angle spinning (CP MAS) ^13^C NMR spectra of both HPB‐COFs (Figure S5, Supporting Information) showed characteristic chemical shifts at ≈159 ppm that were assignable to the carbon signals of the imine bonds. The weak signals ≈18–30 ppm could be assigned to the residual neopentyl groups probably trapped in the pores during the in situ deprotection and polymerization process, which is similar to the previous reported examples.^[^ ^16d^ ^]^ This result was also in line with the stretching vibrations of residue alkyl groups at ≈2900–3000 cm^–1^ in the FT‐IR spectra. Other chemical shifts could be reasonably assigned to the aromatic carbons. Elemental analysis suggested the experimental contents of C, H, and N elements of both HPB‐COFs were close to their theoretical values (Table S1, Supporting Information); it also indicated the chemical composition of 1,2,4‐HPB‐COF was nearly identical to that of 1,3,5‐HPB‐COF, consistent with their isomeric structures. The thermogravimetric analysis showed both HPB‐COFs still maintained 95% of their initial weights up to 550 °C (Figure S6, Supporting Information), which suggests excellent thermal stabilities.

**Figure 2:**
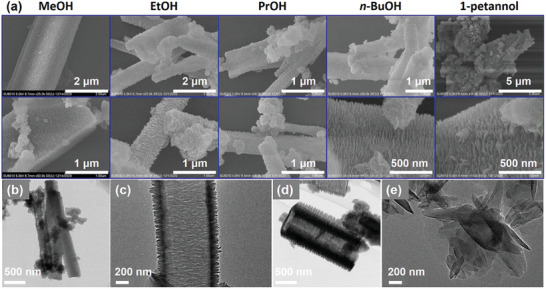
a) SEM images of 1,2,4‐HPB‐COF synthesized from different alcohols with different magnification. b,c) TEM images of 1,2,4‐HPB‐COF at different scale bars. d,e) TEM images of 1,3,5‐HPB‐COF at different scale bars.

The morphologies of both 1,2,4‐HPB‐COF and 1,3,5‐HPB‐COF synthesized from different solvents were investigated through scanning electron microscopy (SEM) and transmission electron microscopy (TEM). **Figure** **2**a and Figure S7 (Supporting Information) showcased both HPB‐COFs synthesized from alcohols exhibited submicrometer‐sized rod‐like structures under low magnification. Interestingly, high‐magnification SEM images revealed these rod‐like structures were actually assembled by highly oriented nanoflakes, which was similar to the metastructured COFs reported before.^[^ ^20^ ^]^ The rod‐like morphology could be delaminated to become random nanosheets upon grounding (Figure S8, Supporting Information). TEM images further confirmed the long‐range ordered rod‐like morphologies were assembled with nanoflakes (Figure 2b–e). These results indicated that the morphologies of HPB‐COFs were not affected by solvent types and both 1,2,4‐HPB‐COF and 1,3,5‐HPB‐COF exhibited similar rod‐like morphologies when synthesized from the same solvents.

**Figure 3:**
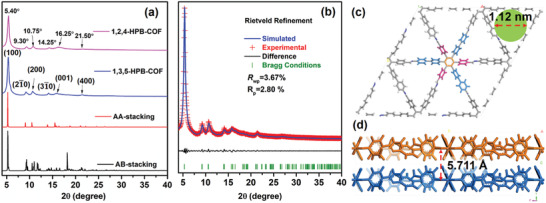
a) Comparison of experimental PXRD patterns of 1,2,4‐HPB‐COF with those of 1,3,5‐HPB‐COF as well as the results of simulated AA‐stacking and AB‐stacking of 1,3,5‐HPB‐COF. b) The experimental PXRD and Rietveld refined XRD patterns of 1,3,5‐HPB‐COF. c) Top view of simulated AA‐stacking mode for 1,3,5‐HPB‐COF (the theoretical pore size is ≈1.12 nm). d) Side view of simulated AA‐stacking mode for 1,3,5‐HPB‐COF (the interlayer distance is ≈5.711 Å).

To resolve the crystalline structures of these two HPB‐COFs, the PXRD patterns of 1,2,4‐HPB‐COF and 1,3,5‐HPB‐COF were compared. **Figure** **3**a indicated two HPB‐COFs showcased almost the same diffraction patterns with identical peak positions. It is difficult to directly simulate the triangular crystal structure of 1,2,4‐HPB‐COF using Materials Studio software due to the desymmetry of 1,2,4‐HPB‐NH_2_ and disorder arrangement of —C═N— bonds in 1,2,4‐HPB‐COF (Figure 1; Figure S1, Supporting Information). Nevertheless, 1,3,5‐HPB‐COF could be readily simulated owing to the *C_3_‐*symmetry of 1,3,5‐HPB‐NH_2_. As shown in Figure 3a, on the basis of the triangular AA stacking mode, the theoretical XRD pattern could reproduce the experimental PXRD result of 1,3,5‐HPB‐COF very well, and the peak positions at ≈5.40°, 9.30°, 10.75°, 14.25°, 16.25°, and 21.50° were attributed to the (100), (2$\bar{1}$0), (200), (3$\bar{1}$0), (001), and (400) facets, respectively. Meanwhile, the six propeller‐like peripheral phenyl units are uniformly distributed around the central benzene core with dihedral angles of ≈66° (Figure S9, Supporting Information). Therefore, it results in a loose stacking mode and a large interlamellar distance of 5.711 Å for 1,3,5‐HPB‐COF (Figure 3d). Interestingly, the experimental PXRD profile of 1,2,4‐HPB‐COF could also match well with the simulated XRD pattern of the triangular 1,3,5‐HPB‐COF in both peak positions and relative intensities. In contrast, the simulated XRD profiles of staggered (AB) stacking largely deviate from the experimental patterns of the two HPB‐COFs (Figure 3a).

Furthermore, the skeleton of 1,3,5‐HPB‐COF was validated by means of Rietveld refinement on the program Topas v5. The refined PXRD profile was in good agreement with the experimental result of 1,3,5‐HPB‐COF and the unit cell parameters were determined as *a* = 1.9296 nm, *b* = 1.9293 nm, *c* = 0.5661, *α* = 90.0009°, *β* = 89.9986°, and *γ* = 120.7114° with negligible deviations (*R*_wp_ = 3.67%, and *R*_p_ = 2.80%, Figure 3b; Table S3, Supporting Information). To resolve the structure of 1,2,4‐HPB‐COF, we also tried to perform Pawley refinement between the optimal AA stacking of 1,3,5‐HPB‐COF and the experimental PXRD of 1,2,4‐HPB‐COF (Figure S10, Supporting Information). The refined PXRD could regenerate the experimental results of 1,2,4‐HPB‐COF with a reasonable difference (*R*_wp_ = 3.64% and *R*_p_ = 2.94%). Therefore, self‐polycondensation of the asymmetric 1,2,4‐HPB‐NH_2_ also produces stable triangular 1,2,4‐HPB‐COF via with unit cell parameters similar as that of 1,3,5‐HPB‐COF. These results also suggest asymmetric “two‐in‐one” building blocks can readily be used to prepare COFs through rational molecular design.

**Figure 4:**
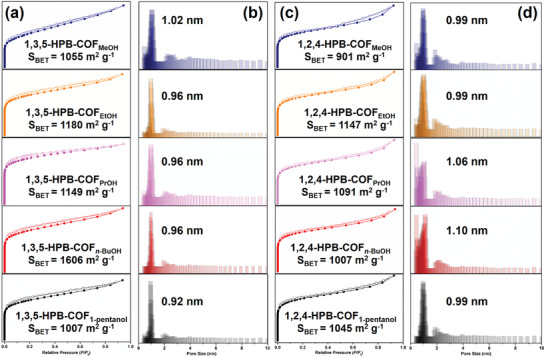
a) N_2_ adsorption‐desorption isotherms and b) pore size distribution profiles of 1,3,5‐HPB‐COF in different solvents; c) N_2_ adsorption‐desorption isotherms and d) pore size distribution profiles of 1,2,4‐HPB‐COF in different solvents.

To further confirm the formation of HPB‐COF with triangular micropores, N_2_ adsorption‐desorption isotherms were measured at 77 K for these HPB‐COFs prepared from five different alcohols. All isotherms of 1,2,4‐HPB‐COF_solvents_ and 1,3,5‐HPB‐COF_solvents_ exhibited type I sorption characteristics^[^ ^21^ ^]^ with a sharp N_2_ uptake under low relative pressure range (*P/P_0_* < 0.05, **Figure** **4**a,c), indicating that all HPB‐COFs are microporous. Most 1,2,4‐HPB‐COF_solvents_ and 1,3,5‐HPB‐COF_solvents_ exhibit similar Brunauer–Emmett–Teller (BET) surface areas of ≈1000 m^2^ g^–1^. According to the nonlocal density functional theory (NLDFT), the pore size distribution (PSD) of the HPB‐COFs was calculated. 1,2,4‐HPB‐COF_solvents_ showed micropores of 0.99–1.10 nm, which were in good agreement with the aperture sizes of 1,3,5‐HPB‐COF_solvents_ and the simulated AA stacking model (1.12 nm, Figure 4b,d). These results again suggested the trigonal porous 1,2,4‐HPB‐COFs were successfully prepared from the asymmetric 1,2,4‐HPB‐NH_2_.

**Figure 5:**
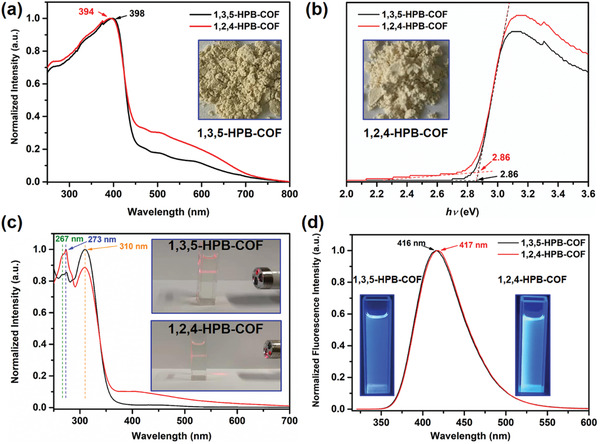
a) Solid‐state UV–vis diffuse reflectance spectra and b) Kubelka–Munk‐transformed reflectance spectra of 1,3,5‐HPB‐COF and 1,2,4‐HPB‐COF. Insert: photographs of 1,3,5‐HPB‐COF in (a) and 1,2,4‐HPB‐COF in (b). c) UV–vis absorption spectra of colloid of 1,3,5‐HPB‐COF and 1,2,4‐HPB‐COF in THF after ultrasound for 6 h. Insert: photographs of 1,3,5‐HPB‐COF and 1,2,4‐HPB‐COF in THF with Tyndall effect. d) Fluorescence spectra of 1,3,5‐HPB‐COF and 1,2,4‐HPB‐COF in THF at 310 nm excitation. Insert: photographs of 1,3,5‐HPB‐COF and 1,2,4‐HPB‐COF in THF under 245 nm ultraviolet radiation.

The positional differences of imine bonds in the 2D COF skeletons may result in different photoelectric properties.^[^ ^22^ ^]^ For example, Seki et al. reported two isomeric 2D *π*‐conjugated COFs with different positional imine linkages, which significantly affect the radical density and conductivity of the two corresponding positional isomeric COFs upon iodine doping.^[^ ^22a^ ^]^ More recently, Brédas et al. reported that the electronic structures of imine‐based isomeric COFs could be associated with imine bond orientations.^[^ ^22b^ ^]^ In consideration of the discrepancy of imine bonds in 1,3,5‐HPB‐COF and 1,2,4‐HPB‐COF, the solid‐state UV‐visible diffuse reflectance spectra were measured. As shown in **Figure** **5**a, the maximum peaks of two HPB‐COFs were nearly identical. Only a small difference in intensities around the region of 450–700 nm was observed. However, the Kubelka–Munk‐transformed reflectance spectra suggested 1,3,5‐HPB‐COF and 1,2,4‐HPB‐COF exhibited the same optical bandgap (Figure 5b). Furthermore, stable colloid solutions could be formed with the obvious Tyndall effect when 1,3,5‐HPB‐COF and 1,2,4‐HPB‐COF were sonicated in THF (Figure 5c). The maximum absorption peaks of two colloid solutions in the UV–vis absorption spectra also were almost identical except in the range of 400–600 nm. These results implied that different imine bonds in 1,3,5‐HPB‐COF and 1,2,4‐HPB‐COF could really lead to a certain difference in photoelectric properties. Whether the slight difference is beneficial for other application performance will be further investigated in our labs. More interestingly, the bulk powders of 1,3,5‐HPB‐COF and 1,2,4‐HPB‐COF was hardly fluorescent as most reported imine COF due to aggregation‐caused quenching phenomenon.^[^ ^23^ ^]^ However, the fluorescence of colloid solutions in THF of two HPB‐COFs were much enhanced through ultrasonic exfoliation to weaken the interlayer C—H···H—C interactions (Figure 5d). The PXRD patterns of 1,3,5‐HPB‐COFs and 1,2,4‐HPB‐COFs were both maintained upon sonication treatments in different solvents (THF and ethanol) at different time intervals (0, 0.5, 3, 6 h). These results suggest that sonication and solvent treatments probably cannot vary the stacking structures of HPB‐COFs, which also indicates the structural stability of the two HPB‐COFs to some extent.

We also tried to further expand the desymmetrization approach by changing the positions of the branches through a co‐polycondensation strategy between asymmetric 1,2,4‐tris(4‐formylphenyl) benzene (1,2,4‐TFB) and asymmetric 1,2,4‐tris(4‐aminophenyl) benzene (1,2,4‐TAB), which change the position of the branch compared with 1,3,5‐triphenyl benzene (Figure S12, Supporting Information). The two asymmetric monomers (1,2,4‐TFB and 1,2,4‐TAB) also were accordingly synthesized and well‐ characterized (Figures S27–S30 and Schemes S6,S7, Supporting Information). Careful screening of conditions is still being attempted in our labs. Given the number of reaction sites and the orientation of the imine bonds, the two monomers can dimerize to form various intermediate fragments similar to “two‐in‐one” type building blocks (Figure S12, Supporting Information). Not all of these fragments can further afford ordered topologies owing to the specific imine bond orientations. As shown in Figures S13 and S14 (Supporting Information), some reasonable representative topologies (1,2,4‐TFB‐TAB‐COFs) are proposed. These results suggest the desymmetrization strategy by changing the positions of the branches is also promising for constructing COFs via the traditional co‐polycondensation method.

## 3 Conclusion

In conclusion, we designed and synthesized two isomeric HPB‐based “two‐in‐one” monomers (1,2,4‐HPB‐NH_2_ and 1,3,5‐HPB‐NH_2_) with neopentyl glycol acetal protecting groups. To our great surprise, the asymmetric monomer 1,2,4‐HPB‐NH_2_ can still form highly crystalline 1,2,4‐HPB‐COFs with nearly identical triangular topological structures as that of 1,3,5‐HPB‐COF synthesized from the symmetric 1,3,5‐HPB‐NH_2_ counterpart. The successfully prepared the triangular 1,2,4‐HPB‐COF demonstrates the feasibility and new possibilities for preparing novel COFs via the branch‐exchange desymmetrization design strategy, which provides new insights into COF design from asymmetric monomers and enhances the diversity of the COF library.

## 4 Experimental Section

### General Procedure of Preparing HPB‐COFs

The “two‐in‐one” type monomers (1,2,4‐HPB‐NH_2_ or 1,3,5‐HPB‐NH_2_) and organic solvent were sequentially added into the Pyrex reaction tube. The mixture was then sonicated and added with acetic acid as a catalyst. After further sonication and three freeze–pump–thaw cycles, the reaction tube was heated at 120 °C. Finally, the precipitated off‐white solid was collected via centrifugation after 3 days. Please refer to Supporting Information for detailed preparation steps of all intermediates, 1,2,4‐HPB‐NH_2_, and 1,3,5‐HPB‐NH_2_.

## Conflict of Interest

The authors declare no conflict of interest.

## Supporting information

Supporting Information

## Data Availability

The data that support the findings of this study are available in the supplementary material of this article.
