# Supplementary material for: Triangular Topological 2D Covalent Organic Frameworks Constructed via Symmetric or Asymmetric “Two‐in‐One” Type Monomers
Source: Adv Sci (Weinh). 2022 Feb 10;9(19):2105517. doi: 10.1002/advs.202105517 (PMC9259724; doi:10.1002/advs.202105517)
Supplement: Supplementary file 1 — Supporting Information [file ADVS-9-2105517-s001.pdf]

ADVANCED SCIENCE
Open Access

Supporting Information

for *Adv. Sci.*, DOI 10.1002/advs.202105517

Triangular Topological 2D Covalent Organic Frameworks Constructed via Symmetric or Asymmetric “Two-in-One” Type Monomers

Weiben Chen, Pei Chen, Dan Chen, Yi Liu, Guang Zhang, Lei Wang and Long Chen**

WILEY-VCH

ADVANCED SCIENCE
Open Access

Supporting Information

for *Adv. Sci.*, DOI: 10.1002/advs.202105517

Triangular Topological 2D Covalent Organic Frameworks Constructed via Symmetric or Asymmetric "Two-in-one" Type Monomers

Weiben Chen, Pei Chen, Dan Chen, Yi Liu Guang Zhang, Lei Wang and Long Chen**

Supporting Information

Triangular Topological 2D Covalent Organic Frameworks Constructed via Symmetric or Asymmetric "Two-in-one" Type Monomers

Weiben Chen, Pei Chen, Dan Chen, Yi Liu Guang Zhang, Lei Wang and Long Chen**

1. Materials and Characterization

4-Ethynylbenzaldehyde, *p*-bromoiodibenzene, Copper(I) Iodide (CuI), $Pd(PPh_3)_2Cl_2$, potassium carbonate, $Pd(PPh_3)_4$, toluene, *N*,*N*-dimethylformamide (DMF), 6 M AcOH (aq.), hydrochloric acid, NH_4Cl, Triethylamine, tetrahydrofuran, anhydrous $MgSO_4$, *p*-benzenesulfonic acid, methanol, ethanol, propanol, *n*-butanol, 1-pentanol, dioxane, ethyl acetate, neopentyl glycol, acetone, dichloromethane, *etc*., the above experimental reagents were directly purchased from Shanghai Titan Scientific Co., Ltd., and were used without further purification.

^{1}H NMR and ^{13}C NMR spectra of all monomers were measured on a Bruker Advance III-400 MHz NMR apparatus. Solid state ^{13}C CP/MAS NMR spectra were measured on a JEOL JNM ECZ600R (600 MHz) solid state NMR instrument. Fourier Transform Infrared (FT-IR) spectra were measured on a Bruker Alpha spectrometer with a range of 400-4000 cm^{-1}. MALDI-TOF spectrometry measurements were performed on the Bruker Autoflex Speed TOF/TOF mass spectrometer. The elemental analysis was measured on an Elementar Model Vario Micro analyzer. Thermogravimetric analysis was measured on a Netzsch TG 209F3 thermogravimetric analyzer in a nitrogen atmosphere; Test rate: 10 °C/min; Test range: room temperature to 800 °C. The Powder X-ray diffraction (PXRD) patterns were recorded on X-ray diffractometer RIGAKU SMARTLAB9KW (unless noted in caption) or DX-27mini X-Ray diffractometer with a Cu-target tube and a graphite monochromator. The light source is a copper target. The theoretical structures of COFs were simulated on Accelrys Materials Studio software. Transmission electron microscope (TEM) and high-resolution transmission electron microscope (HR-TEM) were measured on FEI Model Tecani 20 microscope and JEOL Model JSM-2100F microscope, respectively. Field-emission scanning electron microscope (FE-SEM) images were measured on a JEOL Model JSM-6700 microscope with an acceleration voltage of 5.0 kV. Nitrogen adsorption/desorption isotherms were measured on the BEL Japan Inc. Model Belsorp-Max specific surface analyzer. Before measurement, the samples were heated in a vacuum at 120 °C for 3 hours. The pore size distribution was obtained by isothermal adsorption curves and non-local density functional theory (NLDFT). The Rietveld refinement of 1,3,5-HPB-COF was performed using the Topas v5. Firstly, the Pawley refinement were performed to define the space group and unit cell parameters. Then, the Rietveld refinement were conducted to the structure of 1,3,5-HPB-COF, in which the background, peak profiles, atomic coordinate, unit-cell parameters, , preferred orientation and global isotropic temperature factors were optimized step by step to meet good agreement between the calculated and the experimental powder diffraction patterns (Y-B. Zhang et al., *J. Am. Chem. Soc.* **2019**, *141*, 3298; C. Wang, et al., *Angew. Chem. Int. Ed.* **2020**, *59*, 3624).

2. Synthetic Routes

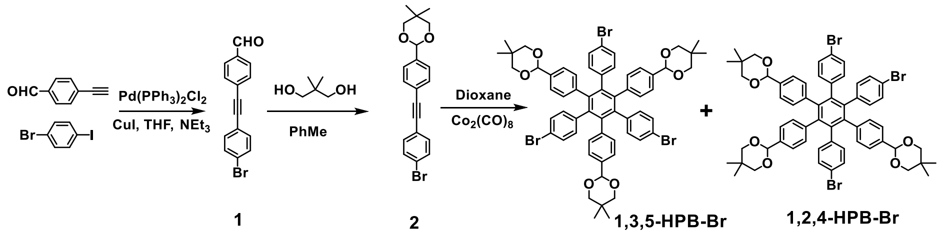

Scheme S1. Synthetic routes of intermediates (**1**, **2**, isomer **1,3,5-HPB-Br** and isomer **1,2,4-HPB-Br**).

Preparation of 4-((4-bromophenyl)ethynyl)benzaldehyde (1) : To a mixture of THF (6 mL) and Et_3N (15 mL) was added 4-ethynylbenzaldehyde (1 g, 7.68 mmol), *p*-bromoiodobenzene (2.24 g, 7.86 mmol), CuI (40 mg, 0.21 mmol) and $Pd(PPh_3)_2Cl_2$ (107 mg, 0.15 mmol). The mixed solution was deoxygenized with argon gas for 30 min. The reaction mixture was stirring at 70 °C for 24 h under argon and cooled down to room temperature. The saturated NH_4Cl aqueous solution was added to quench the reaction. Eethyl acetate (EA) was used to extract product. The organic phase was combined, dried with anhydrous $MgSO_4$ and concentrated under vacuum. Finally, the residue was purified by silica gel column chromatography, affording of 4-((4-bromophenyl)ethynyl)benzaldehyde (**1**) as white powders (1.75 g, yield: 80%). ^{1}H NMR (298 K, 400 MHz, $CDCl_3$) δ (ppm): 10.03 (s, 1H), 7.87 (d, *J* = 8.0 Hz, 2H), 7.67 (d, *J* = 8.0 Hz, 2H), 7.52 (d, *J* = 8.0 Hz, 2H), 7.42 (d, *J* = 8.0 Hz, 2H). ^{13}C NMR ($CDCl_3$, 100 MHz) δ (ppm) = 191.48, 135.56, 133.20, 132.82, 131.82, 129.66, 129.21, 123.37, 92.28, 89.58.

Preparation of 2-(4-((4-bromophenyl)ethynyl)phenyl)-5,5-dimethyl-1,3-dioxane (2): Compounds **1** (570 mg, 2 mmol), neopentyl glycol (1.39 g, 6 mmol) and *p*-toluenesulfonic acid (38 mg, 0.22 mmol) were added to a 50 mL of two-neck round-bottom flask. The reaction mixture was firstly deoxygenized with argon for three times, and then 15 mL of toluene was added. The reaction mixture was heated 115 °C for 12 h under argon and cooled to room temperature. Toluene was removed under vacuum and then the residue was purified by silica gel column chromatography, affording of 2-(4-((4-bromophenyl)ethynyl)phenyl)-5,5-dimethyl-1,3-dioxane (**2**) as a white solid (670 mg, yield: 90%). ^{1}H NMR (298 K, 400 MHz, $CDCl_3$) δ (ppm): 7.50 (m, 6H), 7.39 (m, 2H), 3.75 (d, *J* = 8.0 Hz, 2H), 3.66 (d, *J* = 8.0 Hz, 2H), 3.53 (s, 1H), 1.30 (s, 1H), 0.81 (s, 1H). ^{13}C NMR ($CDCl_3$, 100 MHz) δ (ppm) = 138.66, 133.06, 131.62, 131.58, 126.27, 123.39, 122.53, 122.16, 101.23, 90.39, 88.60, 30.30, 23.07, 21.91.

Preparation of isomer 1,3,5-HPB-Br and isomer 1,2,4-HPB-Br: Isomers **1,3,5-HPB-Br** and **1,2,4-HPB-Br** were prepared in one pot with compound **2**. Compound **2** (446 mg, 1.2 mmol) and octacarbonyldicobalt ($Co_2(CO)_8$) (82 mg, 0.48 mmol) were added to the microwave reaction tube, followed by adding 9 mL of dioxane solvent. The reaction tube was heated at 160 °C and 200 W power for 2 h. After cooling to room temperature, dioxane was removed under vacuum and then the residue was purified by silica gel column chromatography (Eluent: DCM/PE = 6:1 to pure DCM), affording **1,3,5-HPB-Br** (51 mg, yield: 11%) and **1,2,4-HPB-Br** (187 mg, yield: 42%), respectively. ^{1}H NMR of isomer **1,3,5-HPB-Br**: (298 K, 400 MHz, $CDCl_3$) δ (ppm): 6.96 (d, *J* = 8.0 Hz, 6H), 6.89 (d, *J* = 8.0 Hz, 6H), 6.69 (d, *J* = 8.0 Hz, 6H), 6.54 (d, *J* = 8.0 Hz, 6H), 5.11 (s, 3H), 3.63 (d, *J* = 8.0 Hz, 6H), 3.48 (d, *J* = 8.0 Hz, 6H), 1.15 (s, 9H), 0.69 (s, 9H); ^{13}C NMR ($CDCl_3$, 100 MHz) δ (ppm) = 140.24, 140.18, 139.52, 138.97, 135.97, 132.79, 131.13, 130.12, 124.89, 119.91, 101.53, 77.55, 30.24, 23.13, 21.95. ^{1}H NMR of isomer **1,2,4-HPB-Br**: (298 K, 400 MHz, $CDCl_3$) δ (ppm): 6.99 (m, 12H), 6.78 (m, 6H), 6.61 (m, 6H), 5.17 (m, 3H), 3.69 (m, 6H), 3.55 (m, 6H); 1.22 (m, 9H), 0.76 (m, 9H); ^{13}C NMR ($CDCl_3$, 100 MHz) δ (ppm) = 140.44, 140.23, 140.20, 139.93, 139.72, 139.50, 139.29, 139.04, 139.01, 135.99, 135.84, 135.82, 132.89, 132.78, 131.14, 130.19, 130.17, 130.11, 124.92, 124.89, 124.87, 119.93, 119.90, 101.55, 101.52, 77.56, 77.47, 30.25, 30.23, 23.14, 23.09, 21.96.

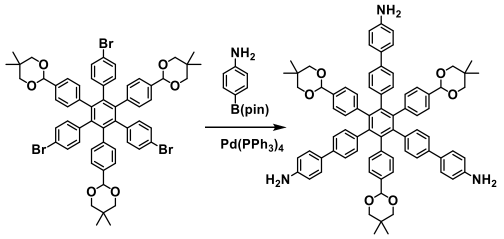

Scheme S2. Synthetic route of symmetric **1,3,5-HPB-NH$_2$**.

Preparation of symmetric 1,3,5-HPB-NH$_2$: Under argon atmosphere, **1,3,5-HPB-Br** (390 mg, 0.35 mmol), 4-(4,4,5,5-tetramethyl-1,3,2-dioxaborolan-2-yl)aniline (375 mg, 1.71 mmol), potassium carbonate (217 mg, 1.57 mmol) and Pd(PPh$_3$)$_4$ (45 mg, 0.038 mmol) were added to 100 mL of single-neck round-bottom flask, The mixed solvents (dioxane/deionized water = 9 mL/1.8 mL) deoxygenated with argon were added successively. The reaction was frozen under liquid nitrogen, degassed and thawed for three times. The reaction was refluxed at 95 °C for 24 h under argon and cooled to room temperature. The reaction mixture was extracted with ethyl acetate for three times, and the organic phase was combined, dried with anhydrous MgSO$_4$ and concentrated under vacuum. The residue was purified by silica gel column chromatography, affording of symmetric **1,3,5-HPB-NH$_2$** as a white solid (270 mg, yield: 67%). ^{1}H NMR (DMSO-d_6, 400 MHz) δ (ppm) = 7.20 (d, J = 8.0 Hz, 6H), 7.06 (d, J = 8.0 Hz, 6H), 6.89 (m, 12H), 6.80 (d, J = 8.0 Hz, 6H), 6.51 (d, J = 8.0 Hz, 6H), 5.17 (s, 6H), 5.08 (s, 6H), 3.50 (d, J = 8.0 Hz, 6H), 3.40 (d, J = 8.0 Hz, 6H), 1.05 (s, 9H),0.63 (s, 9H). ^{13}C NMR (DMSO-d_6, 100 MHz) δ (ppm) = 148.70, 141.14, 140.46, 140.31, 139.64, 137.87, 137.05, 131.08, 127.06, 125.64, 125.18, 123.39, 123.38, 114.55, 103.78, 101.33, 100.00, 79.77, 79.44, 79.11, 76.74, 30.09, 23.14, 21.87. HR-MS (MALDI–TOF): calcd. for $C_{76}H_{53}N_6$ m/z = 1150.57286 [M+H]$^+$, found: m/z =1150.57556 [M+H]$^+$.

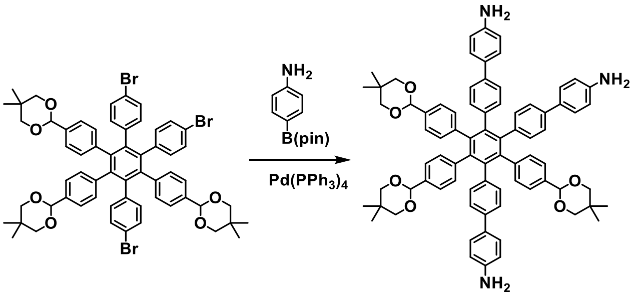

Scheme S3. Synthetic route of asymmetric **1,2,4-HPB-NH$_2$**

Preparation of asymmetric 1,2,4-HPB-NH$_2$: Isomer **1,2,4-HPB-Br** (390 mg, 0.35 mmol), 4-(4,4,5,5-tetramethyl-1,3,2-dioxaborolan-2-yl)aniline (375 mg, 1.71 mmol), potassium carbonate (217 mg, 1.57 mmol) and Pd(PPh$_3$)$_4$ (45 mg, 0.038 mmol) were added to 100 mL of single-neck round-bottom flask, The mixed solvents (dioxane/deionized water = 9 mL/1.8 mL) deoxygenated with argon was added. The reaction was frozen under liquid nitrogen, degassed and thawed for three times. The reaction was refluxed at 95 °C for 24 h under argon. After cooled to room temperature, the reaction was extracted with ethyl acetate for three times, and the organic phase was combined, dried with anhydrous MgSO$_4$ and filtered. The solvents were removed from filtrate and the residue was purified by column chromatography to obtain a white solid. (210 mg, yield: 52%). ^{1}H NMR (DMSO-d_6, 400 MHz) δ (ppm) = 7.27 (m, 6H), 7.14 (d, J = 8.0

Hz, 6H), 6.99 (m, 12H), 6.90 (m, 6H), 6.55 (m, 6H), 5.17 (s, 6H), 5.21 (s, 6H), 3.59 (m, 6H), 3.40 (m, 6H), 1.14 (m, 9H),0.73 (m, 9H). ^{13}C NMR (DMSO-d_6, 100 MHz) δ (ppm) = 148.73, 148.68, 148.65, 141.18, 141.07, 141.05, 140.61, 140.56, 140.44, 140.39, 140.20, 140.07, 137.98, 137.95, 137.85, 137.12, 137.03, 135.89, 135.85, 131.80, 131.07, 127.10, 127.05, 126.72, 126.70, 126.66, 125.23, 123.43, 123.40, 115.98, 115.70, 114.54, 101.31, 76.75, 30.14, 30.11, 23.18, 23.16, 21.88. HR-MS (MALDI–TOF): calcd. for $C_{76}H_{53}N_6$ m/z=1150.57286 $[M+H]^+$, found: m/z =1150.57581 $[M+H]^+$.

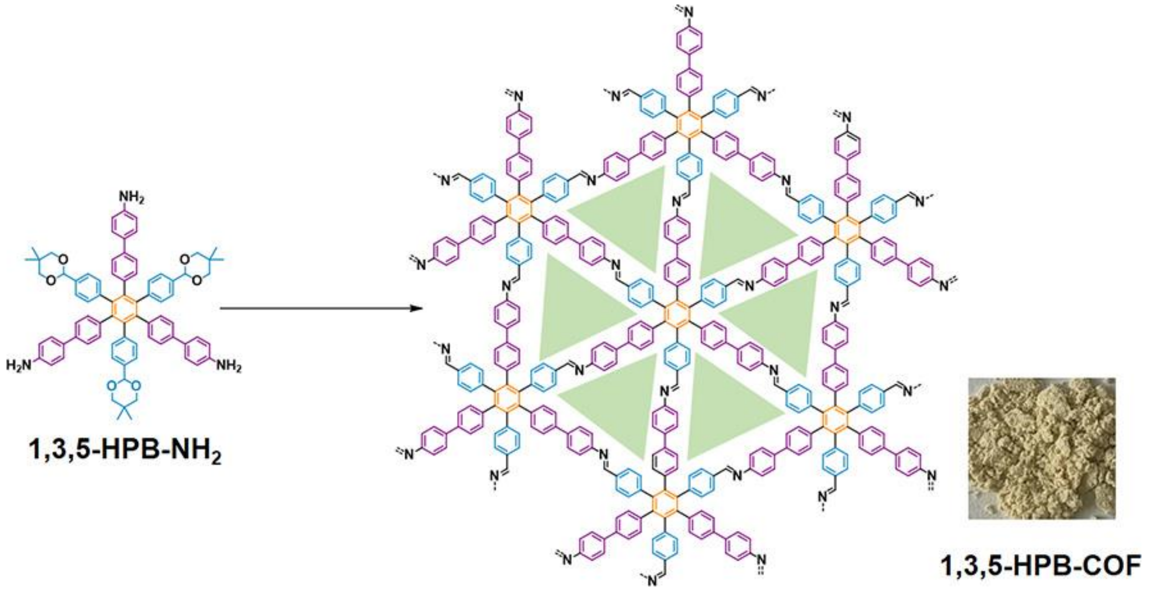

Scheme S4. Synthetic route of symmetric **1,3,5-HPB-COF**

Preparation of symmetric 1,3,5-HPB-COF: The symmetric monomer **1,3,5-HPB-NH$_2$** (30 mg, 0.042 mmol) and organic solvent (1 mL) were successively added into a 10 mL of Pyrex reaction tube. The reaction tube was then ultrasonic for 1 min. Following, 0.3 mL of 6 M acetic acid solution was added. The reaction tube was then ultrasonic for 1 min. The reaction tube was frozen under liquid nitrogen, degassed and thawed for three times. Finally, the reaction tube was placed in an oven at 120 ℃ for three days. After cooled to room temperature, the precipitated gray solid was filtered, and washed with DMF, anhydrous ethanol, acetone and dichloromethane for three times, respectively and dried under vacuum for 24 h (16 mg, yield: 58%).

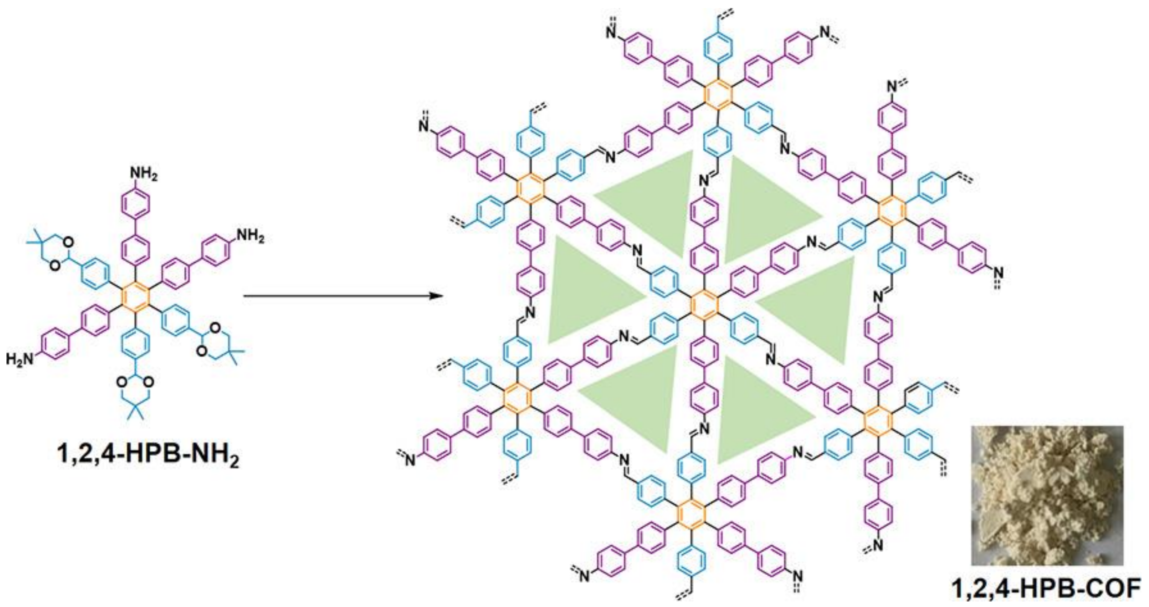

Scheme S5. Synthetic route of asymmetric **1,2,4-HPB-COF**

Preparation of asymmetric 1,2,4-HPB-COF: The asymmetric monomer **1,2,4-HPB-NH$_2$** (30 mg, 0.042 mmol) and organic solvent (1 mL) were successively added into a 10 mL of Pyrex reaction tube. The reaction tube was then ultrasonic for 1 min. Following, 0.3 mL of 6 M acetic acid solution was added. The reaction tube was then ultrasonic for 1 min. The reaction tube was frozen under liquid nitrogen, degassed and thawed for three times. Finally, the reaction tube was placed in an oven at 120 °C for three days. After cooled to room temperature, the precipitated gray solid was filtered, and washed with DMF, anhydrous ethanol, acetone and dichloromethane for three times, respectively. and dried under vacuum for 24 h (23 mg, yield: 84%).

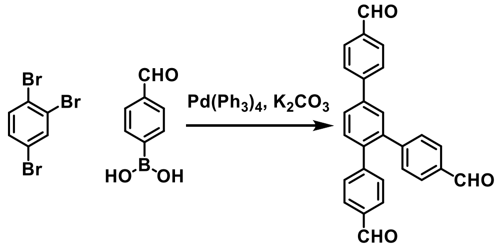

Scheme S6. Synthetic route of asymmetric **1,2,4-TFB**.

Preparation of asymmetric 1,2,4-TFB: Under argon atmosphere, 1,2,4-tribromobenzene (700 mg, 2.22 mmol), (4-formylphenyl)boronic acid (1.344 g, 8.96 mmol), potassium carbonate (1.344 g, 9.72 mmol) and $Pd(PPh_3)_4$ (259 mg, 0.22 mmol) were added to 100 mL of single-neck round-bottom flask, The mixed solvents (dioxane/deionized water = 35 mL/14 mL) deoxygenated with argon was added successively. The reaction was frozen under liquid nitrogen, degassed and thawed for three times. The reaction was refluxed at 100 °C for 24 h under argon and cooled to room temperature. The reaction mixture was extracted with ethyl acetate for three times, and organic phase was combined, dried with anhydrous $MgSO_4$ and concentrated under vacuum. The residue was purified by silica gel column chromatography, affording of asymmetric 1,2,4-TFB as off-white solid (700 mg, yield: 80%). ^{1}H NMR (298 K, 400 MHz, $CDCl_3$) δ (ppm): 10.09 (s, 1H), 10.01 (s, 1H), 9.99 (s, 1H), 8.01 (m, 2H), 7.85 (m, 2H), 7.79 (m, 5H), 7.74 (m, 1H),

7.61 (m, 1H), 7.35 (m, 4H). ^{13}C NMR ($CDCl_3$, 100 MHz) δ (ppm) = 191.80, 191.78, 146.92, 146.59, 145.84, 140.17, 140.05, 139.43, 135.66, 135.04, 134.99, 131.39, 130.52, 130.45, 130.44, 129.69, 129.65, 129.58, 127.76, 127.39.

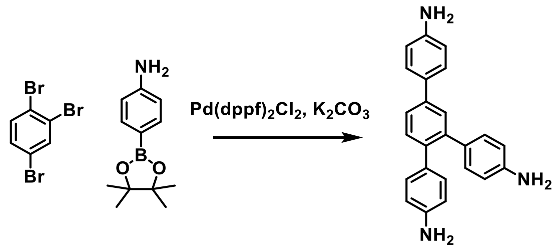

Scheme S7. Synthetic route of asymmetric **1,2,4-TAB**.

Preparation of asymmetric 1,2,4-TAB: Under argon atmosphere, 1,2,4-tribromobenzene (1 g, 3.17 mmol), (4-formylphenyl)boronic acid (2.14 g, 9.77 mmol), potassium carbonate (2.8 g, 20.26 mmol) and 1,1'-Bis(diphenylphosphino)ferrocene]dichloropalladium(II) ($Pd(dppf)_2Cl_2$) (500 mg, 0.68 mmol) were added to 100 mL of single-neck round-bottom flask, The mixed solvents (dioxane/deionized water = 50 mL/10 mL) deoxygenated with argon was added successively. The reaction was frozen under liquid nitrogen, degassed and thawed for three times. The reaction was refluxed at 100 ℃ for 24 h under argon and cooled to room temperature. The reaction was extracted with ethyl acetate for three times, and organic phase was combined, dried with anhydrous $MgSO_4$ and concentrated under vacuum. The residue was purified by silica gel column chromatography, affording of asymmetric 1,2,4-TAB as off-white solid. (1 g, yield: 90%). ^{1}H NMR (298 K, 400 MHz, $CDCl_3$) δ (ppm): 7.50 (m, 3H), 7.39 (m, 1H), 7.00 (m, 3H), 6.76 (m, 2H), 6.57 (m, 3H), 3.64 (s, 6H). ^{13}C NMR ($CDCl_3$, 100 MHz) δ (ppm) = 144.80, 144.70, 140.56, 139.61, 138.36, 132.43, 132.01, 131.25, 130.83, 130.78, 128.51, 127.97, 124.76, 115.43, 114.76.

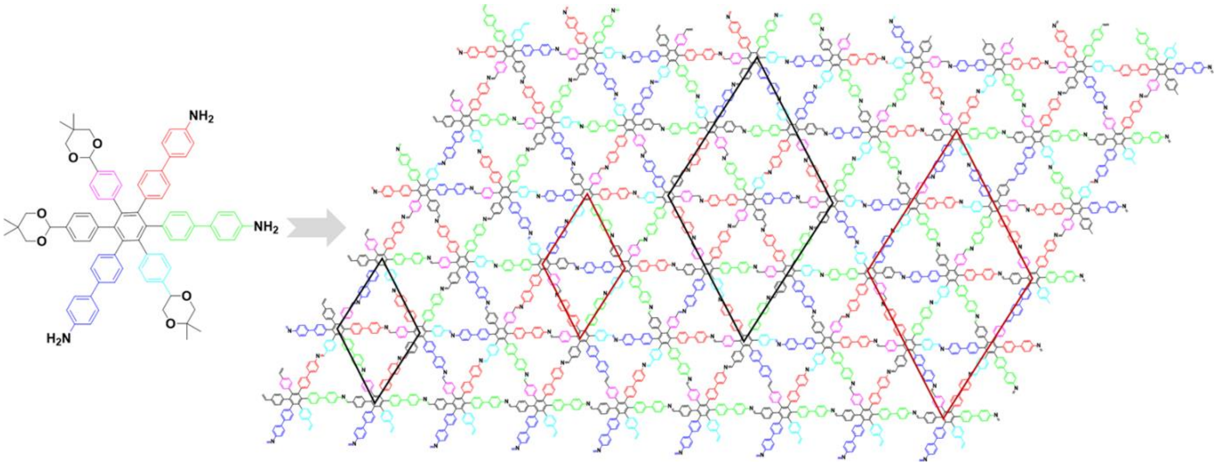

Figure S1. Every branch in 1,2,4-HPB-NH_2 is unique and is labeled by using different colors. Although 1,2,4-HPB-NH_2 is asymmetry and the orientation of imine bond is irregular, 1,2,4-HPB-NH_2 still self-assemble into the topology with triangular pore. We tried to find minimum repeating structure in the triangular topology. As showed in **Figure S1**, the black rhombus represents a repeating structure, which is inconsistent with the red rhombus. The results suggest it is difficult to find a suitable repeating unit cell and to directly simulate the long-range ordering structure of triangular 1,2,4-HPB-COF.

3. Structural Characterization of 1,3,5-HPB-COFs and 1,2,4-HPB-COFs

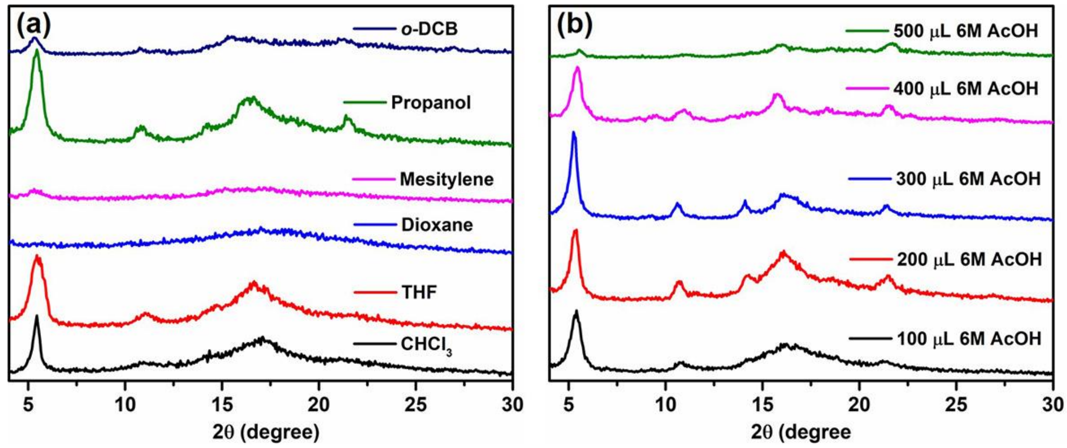

Figure S2. (a) PXRD patterns of 1,2,4-HPB-COFs at different solvents. (b) PXRD patterns of 1,2,4-HPB-COFs using propanol at different amounts of 6M acetic acid. (note: data collected on DX-27 mini X-Ray diffractometer, 600 W).

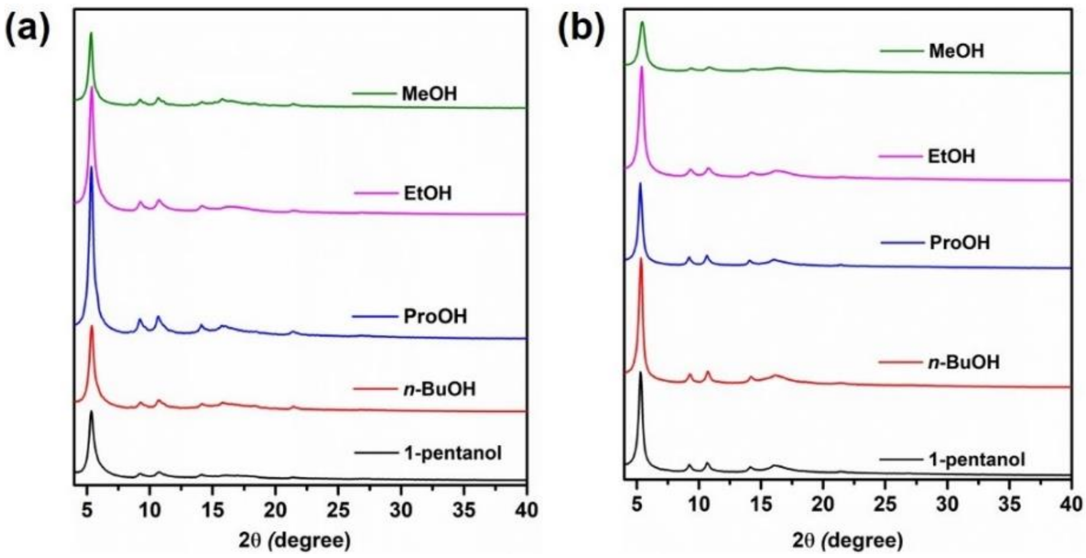

Figure S3. PXRD profiles of (a) 1,3,5-HPB-COF and (b) 1,2,4-HPB-COFs under different solvents for the simple reaction condition screening. (note: data collected on RIGAKU SMARTLAB9KW X-Ray diffractometer, 9000 W).

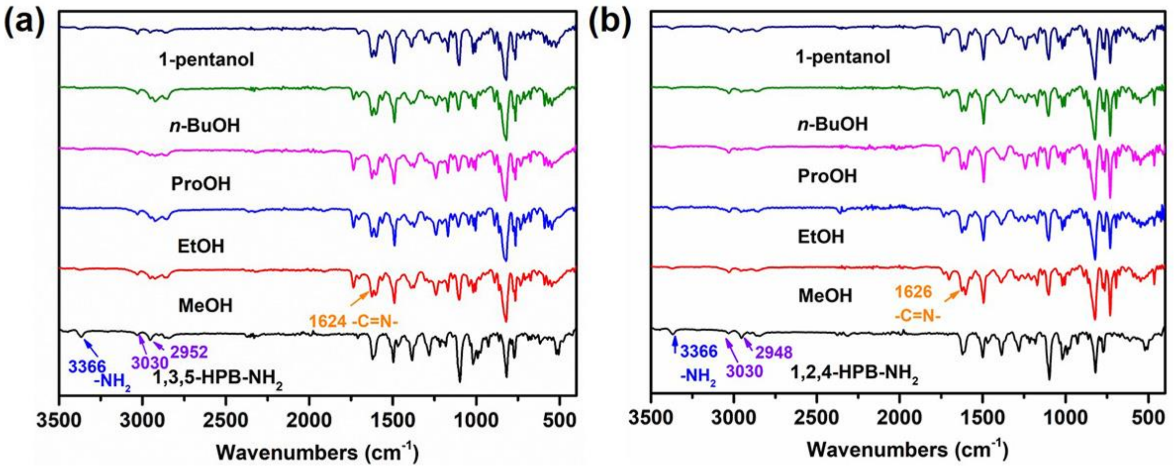

Figure S4. FT-IR spectra of (a) 1,3,5-HPB-COFs and (b) 1,2,4-HPB-COFs under different solvents for the simple reaction condition screening.

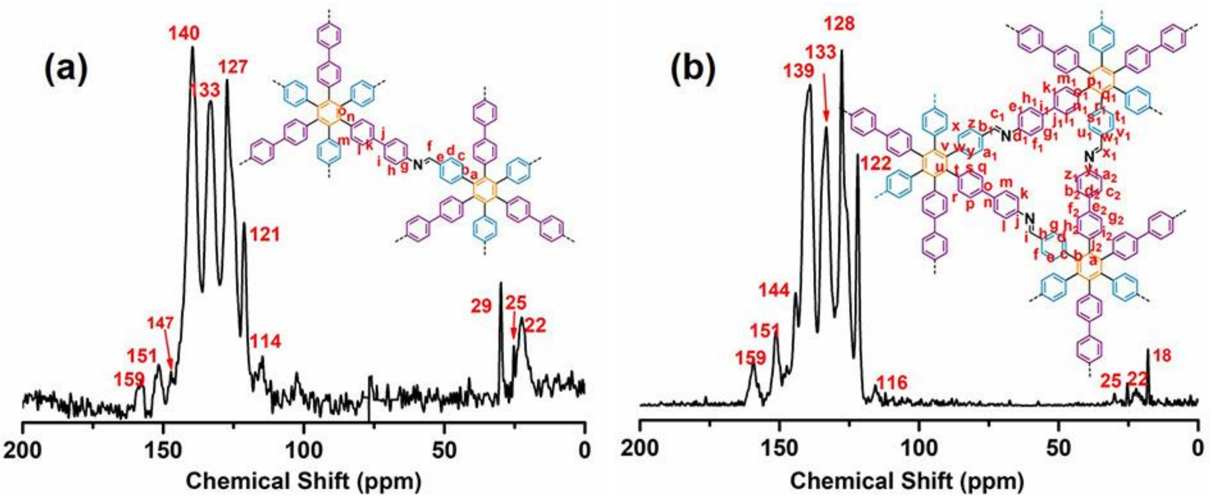

Figure S5. Solid-state ^{13}C-NMR spectral profiles of (a) 1,3,5-HPB-COF and (b) 1,2,4-HPB-COF.

Table S1. Elemental analysis of (a) 1,3,5-HPB-COF and (b) 1,2,4-HPB-COFs.

COFs		C/%	H/%	N/%
**1,3,5-HPB-COF**	Calculated	89.97	5.03	5.00
	Found	81.75	5.01	4.32
**1,2,4-HPB-COF**	Calculated	89.97	5.03	5.00
	Found	81.41	5.99	3.89

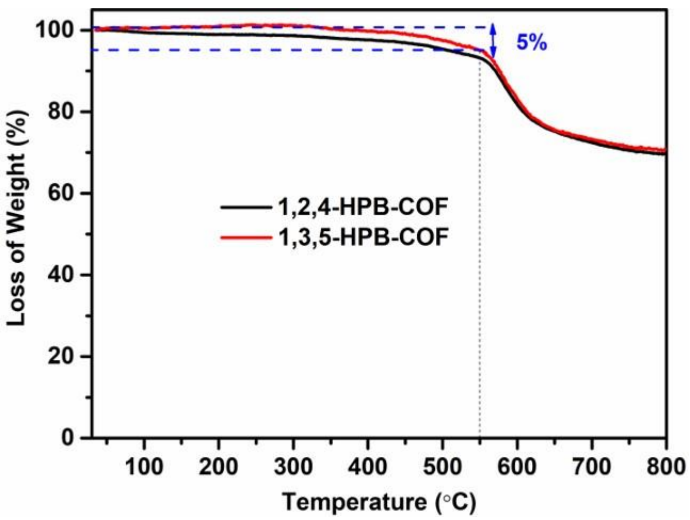

Figure S6. Thermogravimetric analysis of 1,3,5-HPB-COF and 1,2,4-HPB-COF.

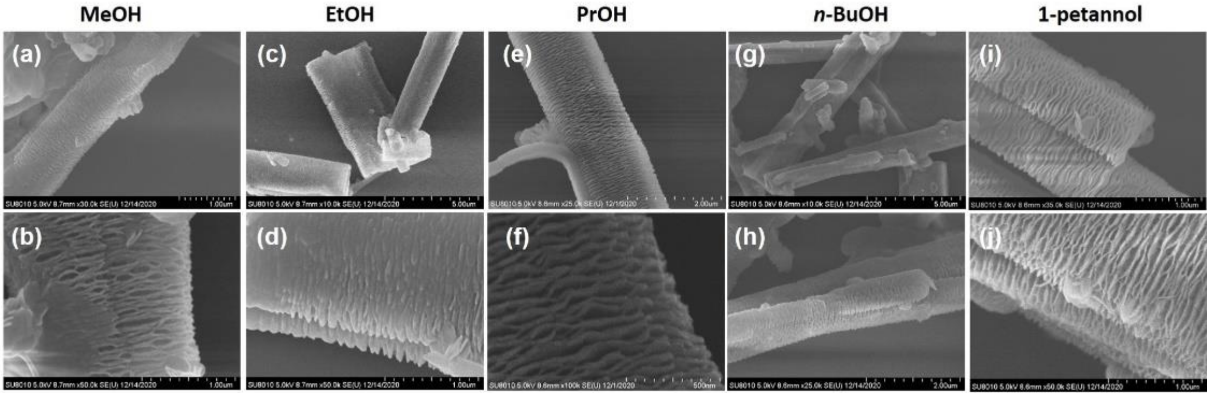

Figure S7. SEM images of (a) 1,3,5-HPB-COFs in (a)(b) MeOH, (c)(d) EtOH, (e)(f) PrOH, (g)(f) *n*-BuOH and (i)(j) 1-pentanol at different scales.

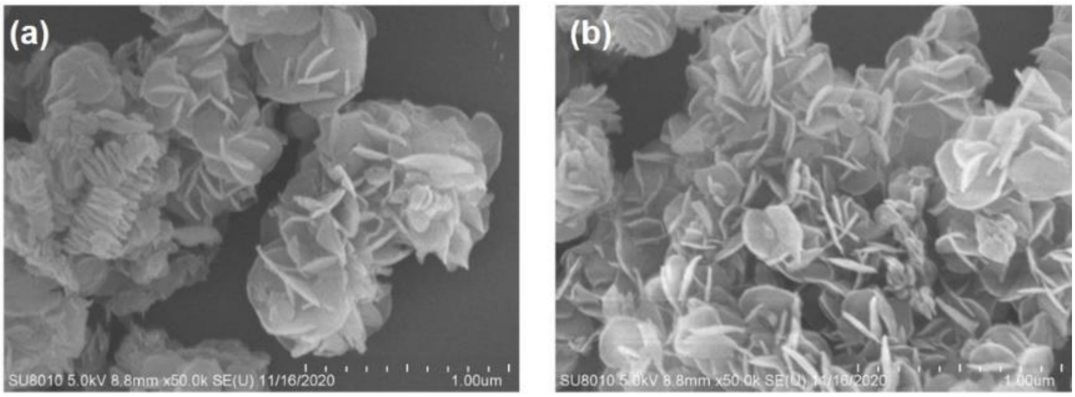

Figure S8. SEM images of (a) and (b) 1,3,5-HPB-COF after grounding.

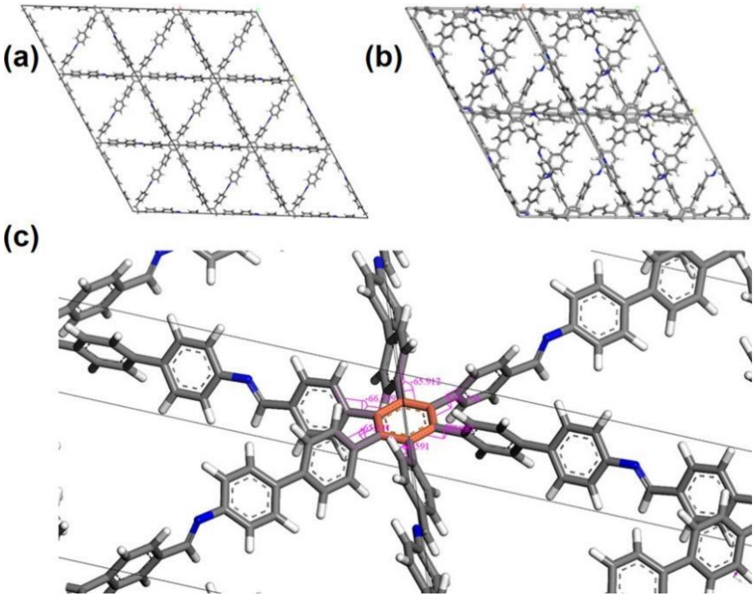

Figure S9. (a) Top view of simulated AA stacking for 1,3,5-HPB-COF; (b) Top view of simulated AB stacking for 1,3,5-HPB-COF; (c) The dihedral angle between the central benzene and the six linked benzene on simulated AA stacking.

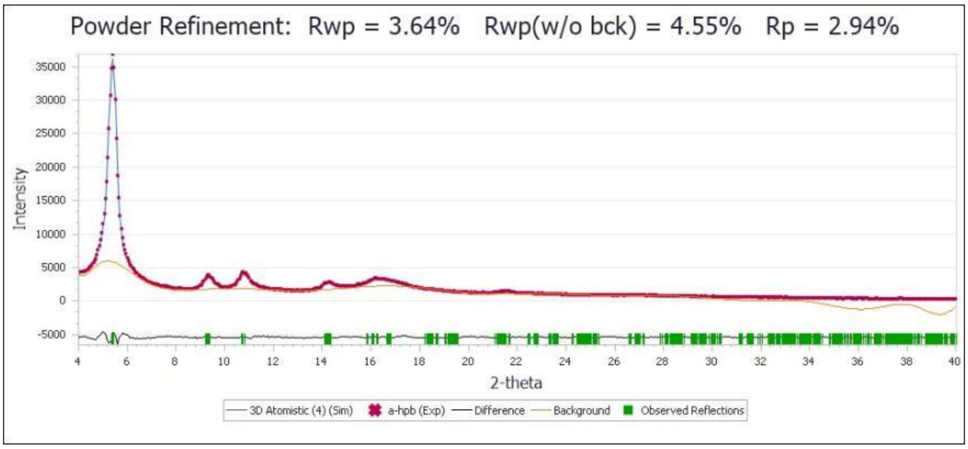

Figure S10. Pawley refinement between the optimal AA stacking of 1,3,5-HPB-COF and the experimental PXRD of 1,2,4-HPB-COF.

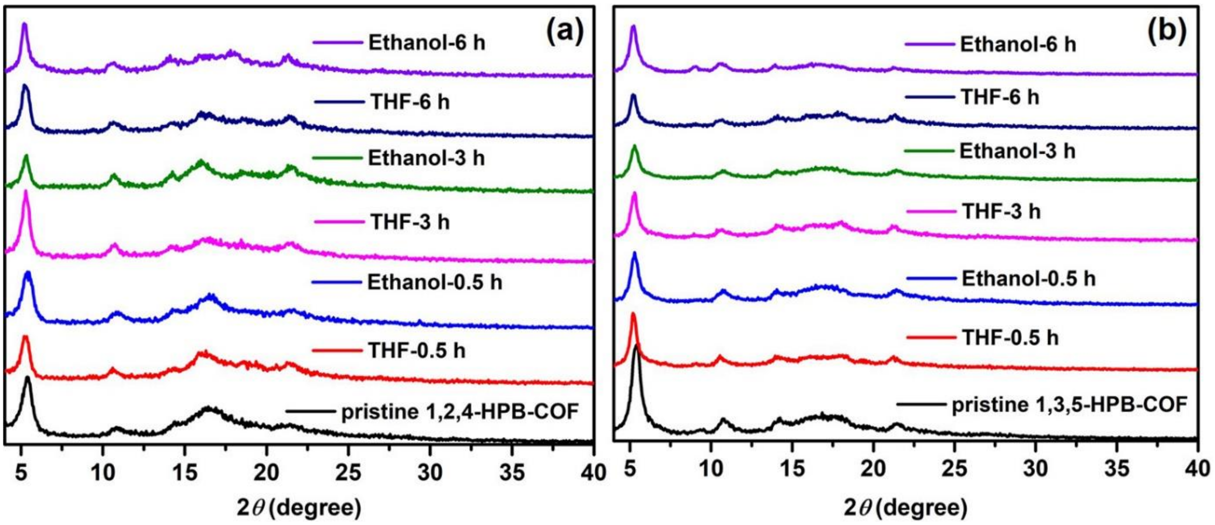

Figure S11. PXRD patterns of 1,2,4-HPB-COFs (a) and 1,3,5-HPB-COFs (b) were measured by applying sonication treatment in different solvents (THF and ethanol) under different times (0 h, 0.5 h, 3 h, 6 h). Note: data collected on DX-27 mini X-Ray diffractometer, 600 W.

4. Design and Characterization of 1,2,4-TFB-TAB-COFs

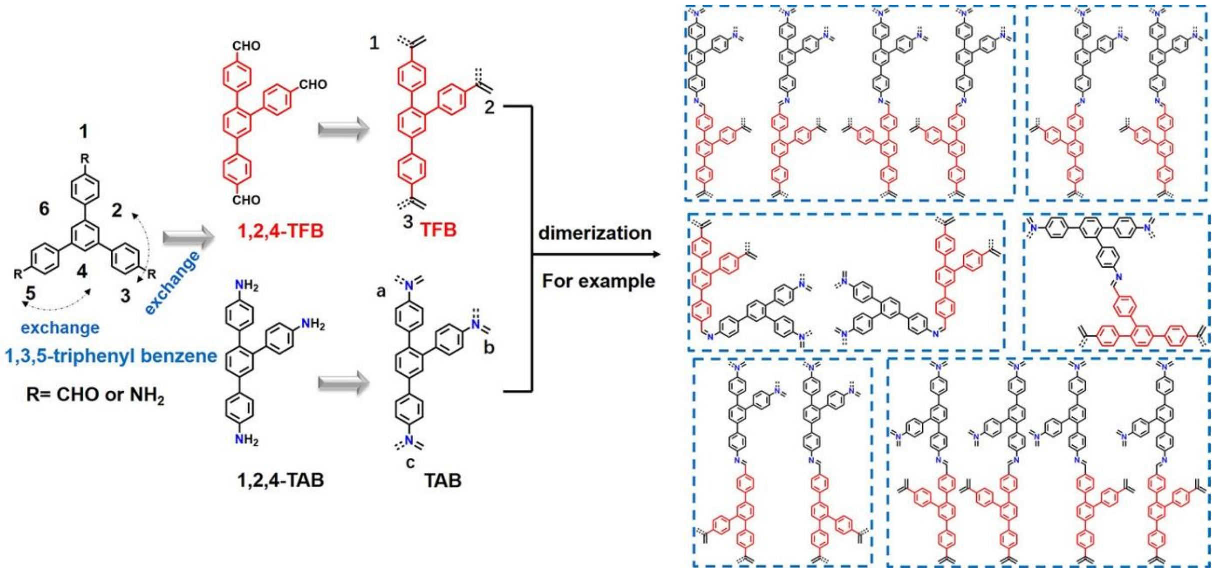

Figure S12. Changing the position of the branch of C_3 symmetric 1,3,5-triphenyl benzene to obtain asymmetric 1,2,4-triphenyl benzene isomers (1,2,4-TFB and 1,2,4-TAB). Note: In order to facilitate drawing structure, 1,2,4-TFB and 1,2,4-TAB are simplified to TFB and TAB in Figure S11. In consideration of the number of reaction sites and the orientation of the imine bonds, the two monomers can dimerize to form various intermediate fragments. As showcased in Figure S11, the representative dimer structures are similar to “two-in-one” building blocks, which will further form more complex frameworks.

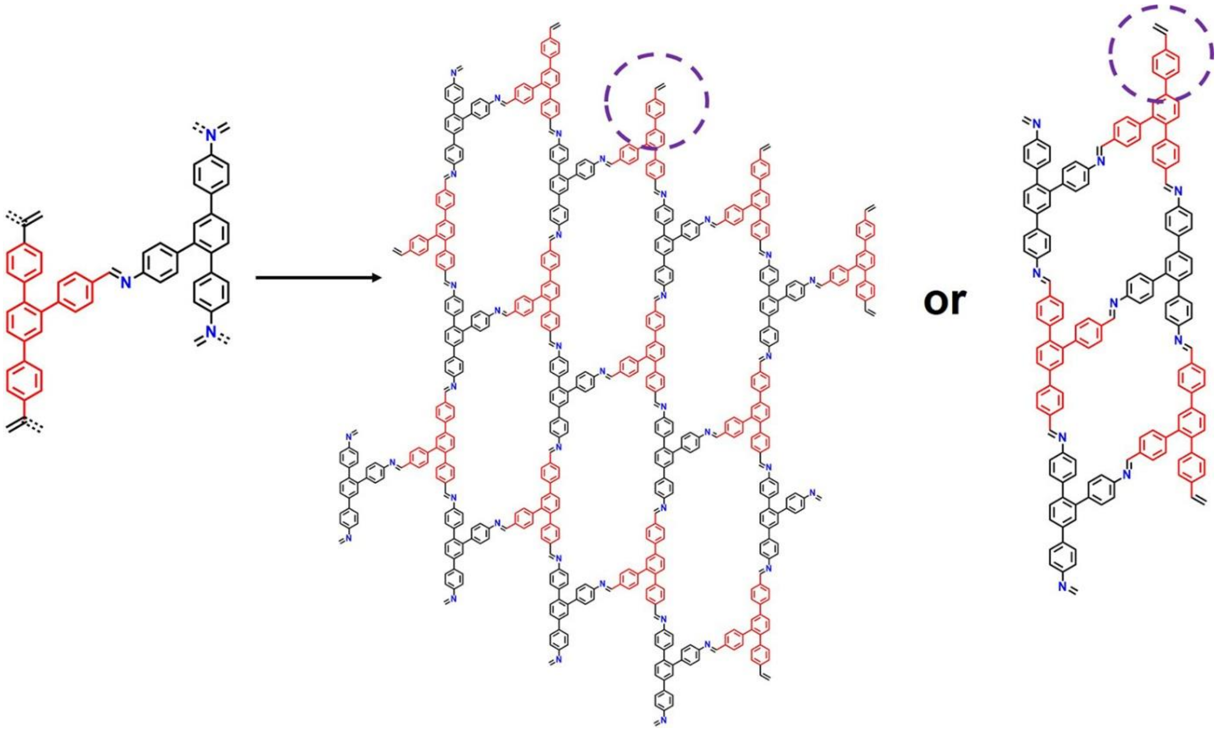

Figure S13. In consideration of the orientation of the imine bonds, the same intermediate fragments can form two different topological structures.

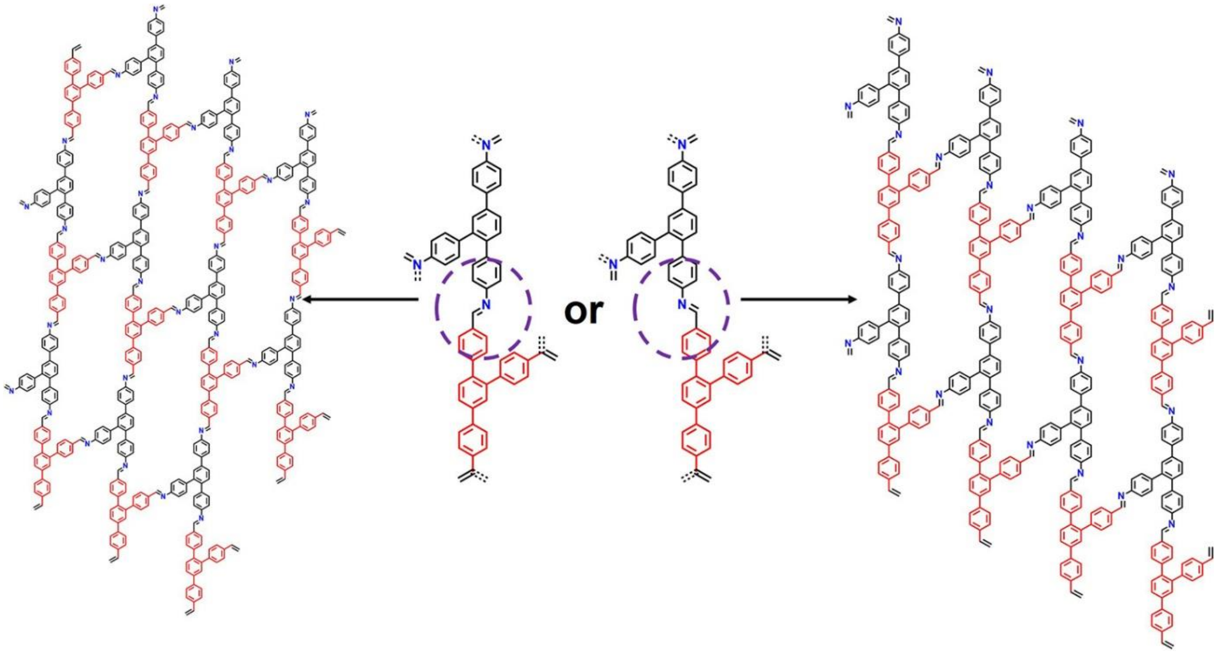

Figure S14. In consideration of the orientation of the imine bonds in the similar intermediate fragments, two quadrilateral topologies with different sizes of aperture are formed. Additionally, as showed in Figure S12 and S13, the intermediate fragments from the different reactive sites can form hexagonal, quadrilateral and rhombic topological structures. Therefore, we predict copolymerization between 1,2,4-TFB and 1,2,4-TAB in different reaction conditions could form various topological structures.

5. Characterization of Intermediates and Monomers

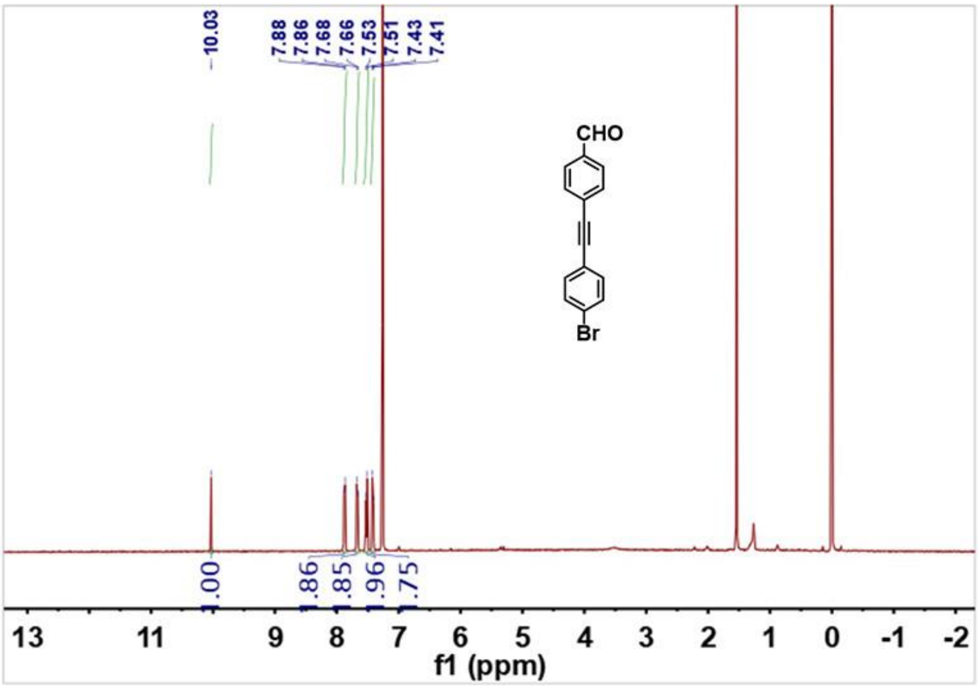

Figure S15. ^{1}H NMR ($CDCl_3$, 400 MHz) spectra of intermediate **1**

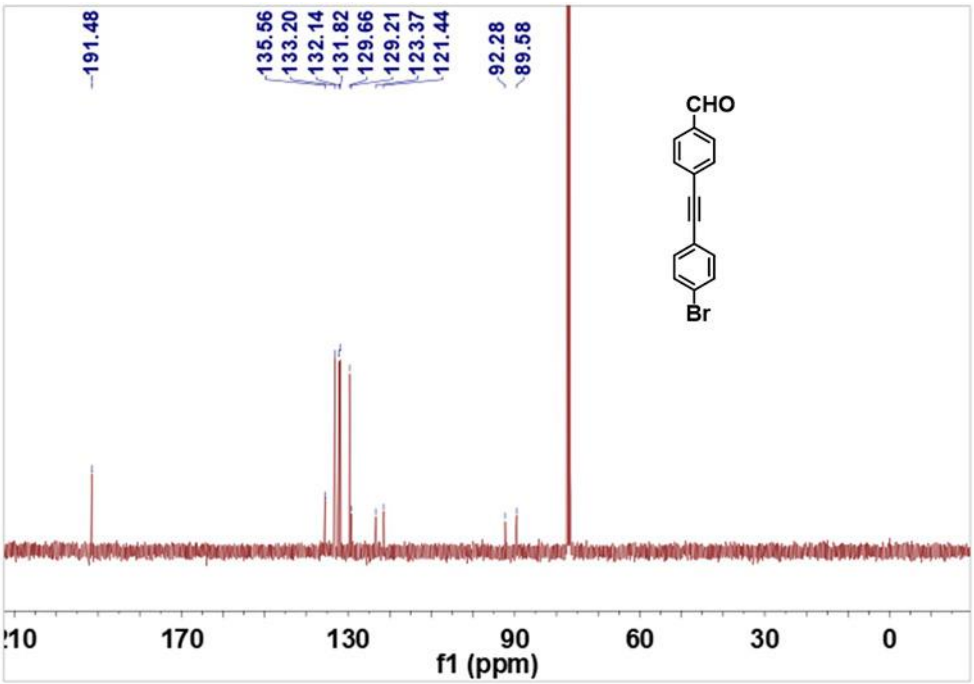

Figure S16. ^{13}C NMR ($CDCl_3$, 100 MHz) spectra of intermediate **1**

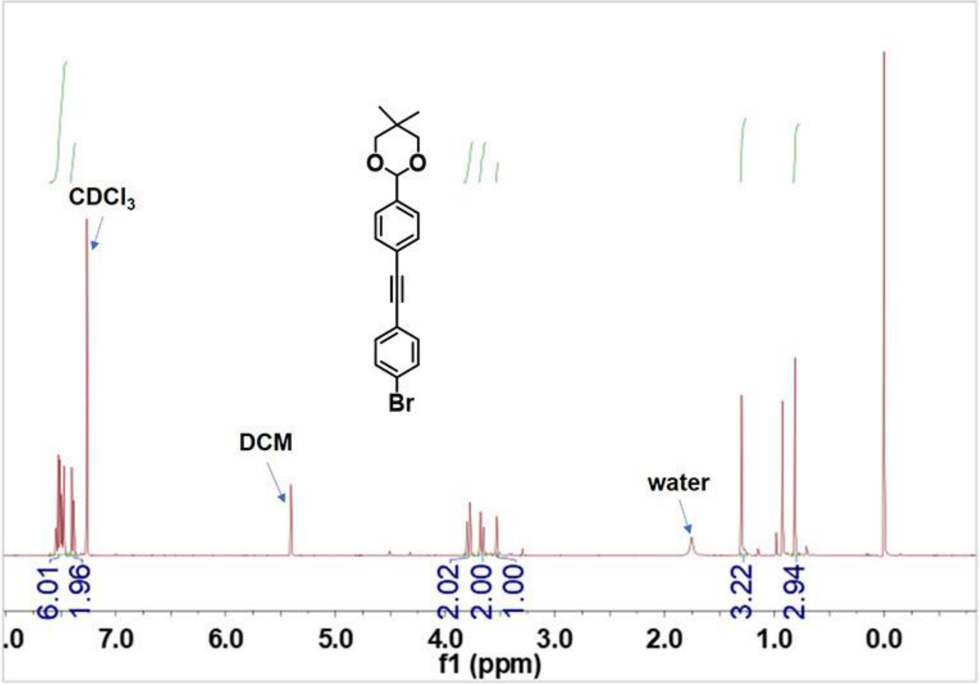

Figure S17. ^{1}H NMR ($CDCl_3$, 400 MHz) spectra of intermediate **2.**

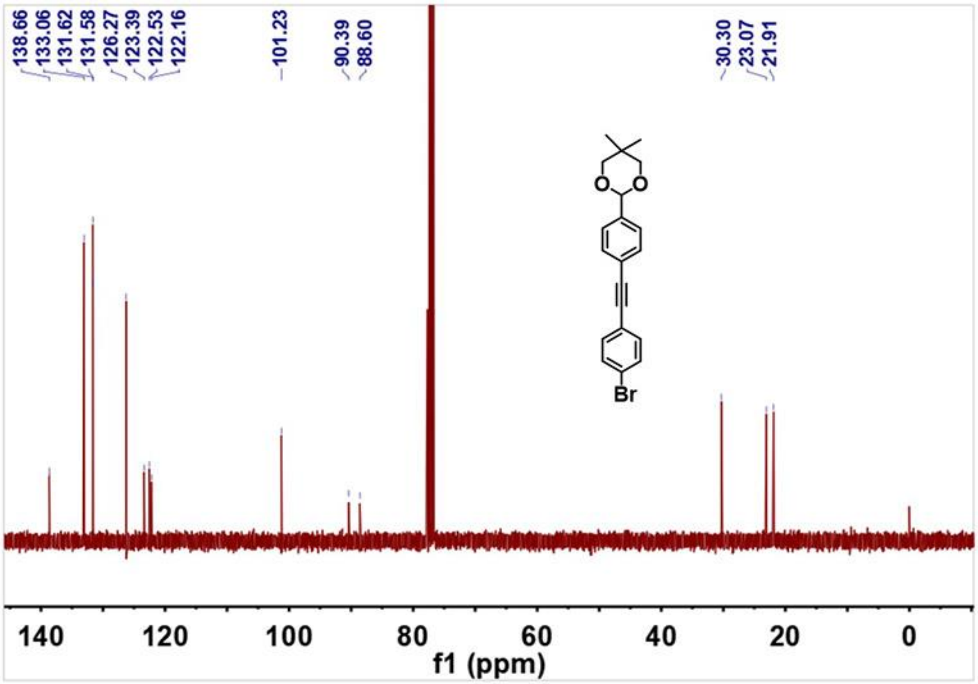

Figure S18. ^{13}C NMR ($CDCl_3$, 100 MHz) spectra of intermediate **2.**

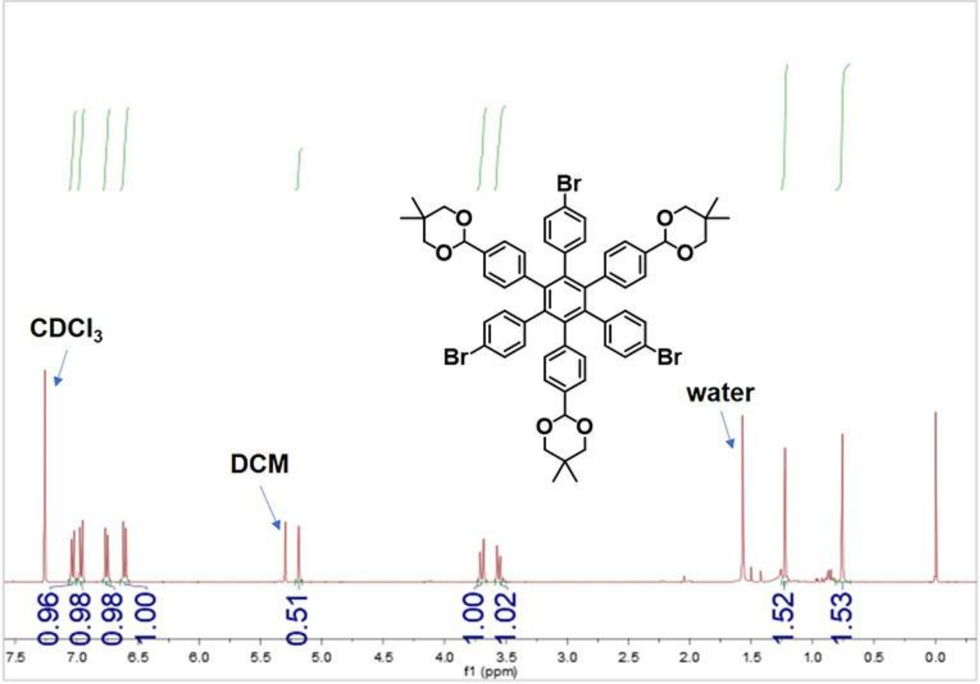

Figure S19. ^{1}H NMR ($CDCl_3$, 400 MHz) spectra of 1,3,5-HPB-Br.

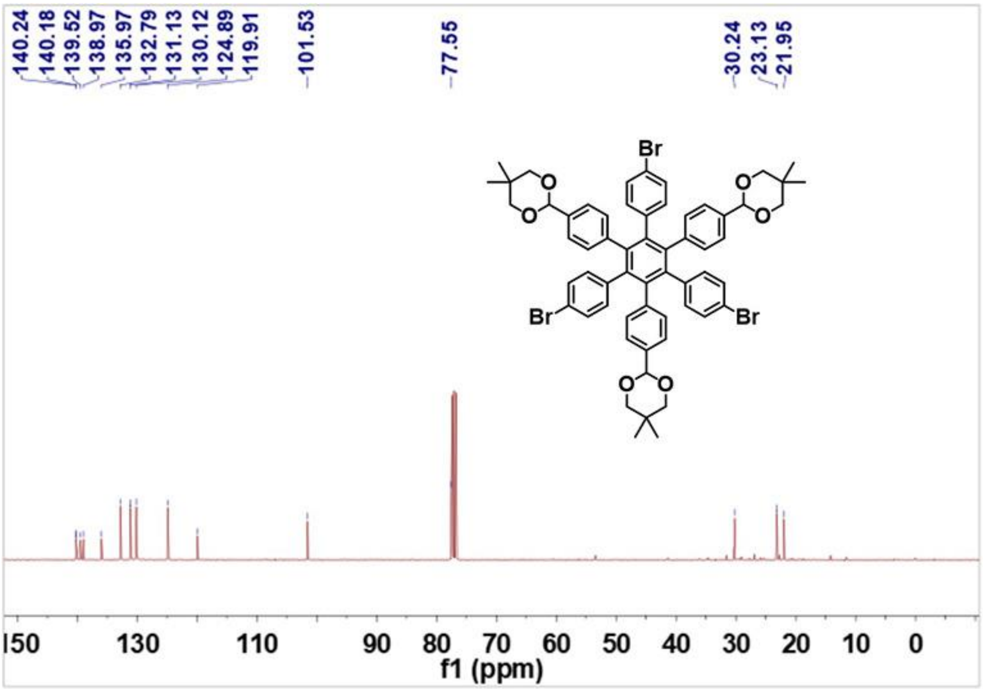

Figure S20. ^{13}C NMR ($CDCl_3$, 100 MHz) spectra of 1,3,5-HPB-Br.

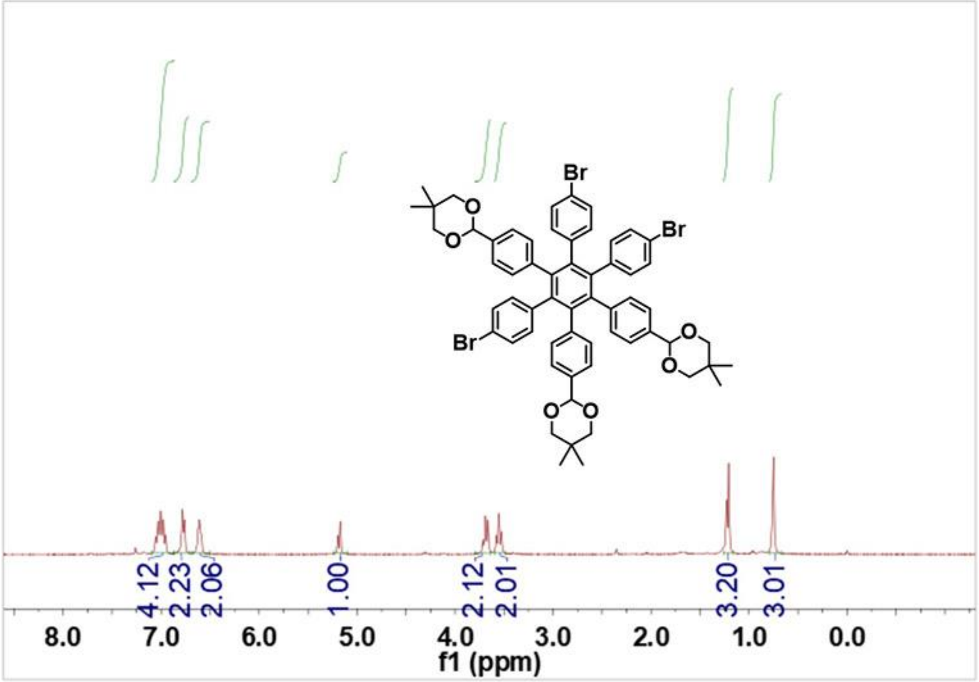

Figure S21. ^{1}H NMR ($CDCl_3$, 400 MHz) spectra of 1,2,4-HPB-Br.

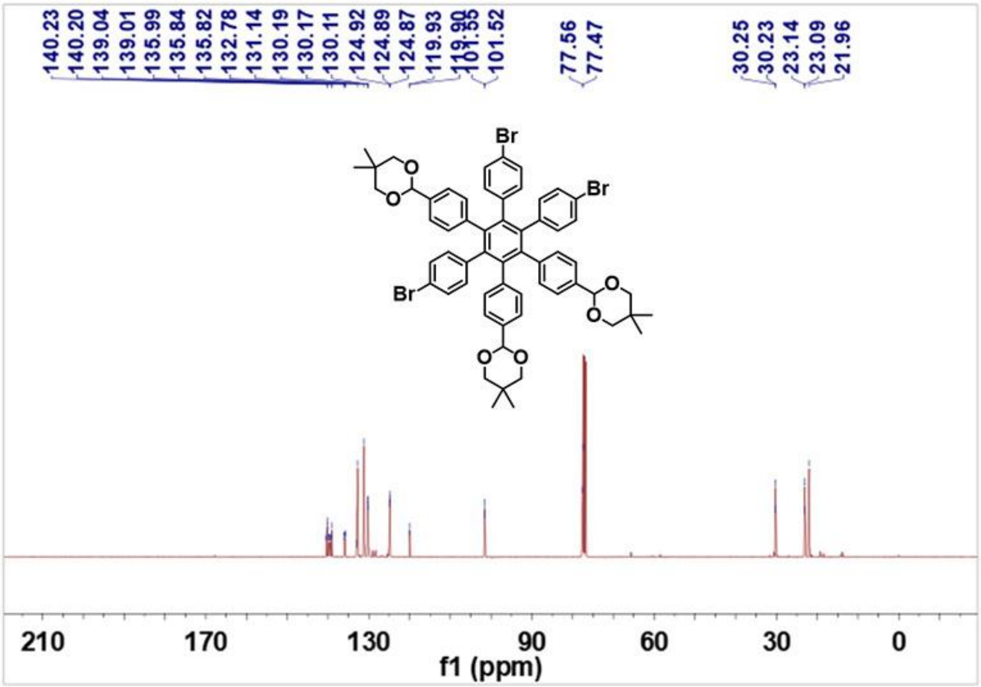

Figure S22. ^{13}C NMR ($CDCl_3$, 100 MHz) spectra of 1,2,4-HPB-Br.

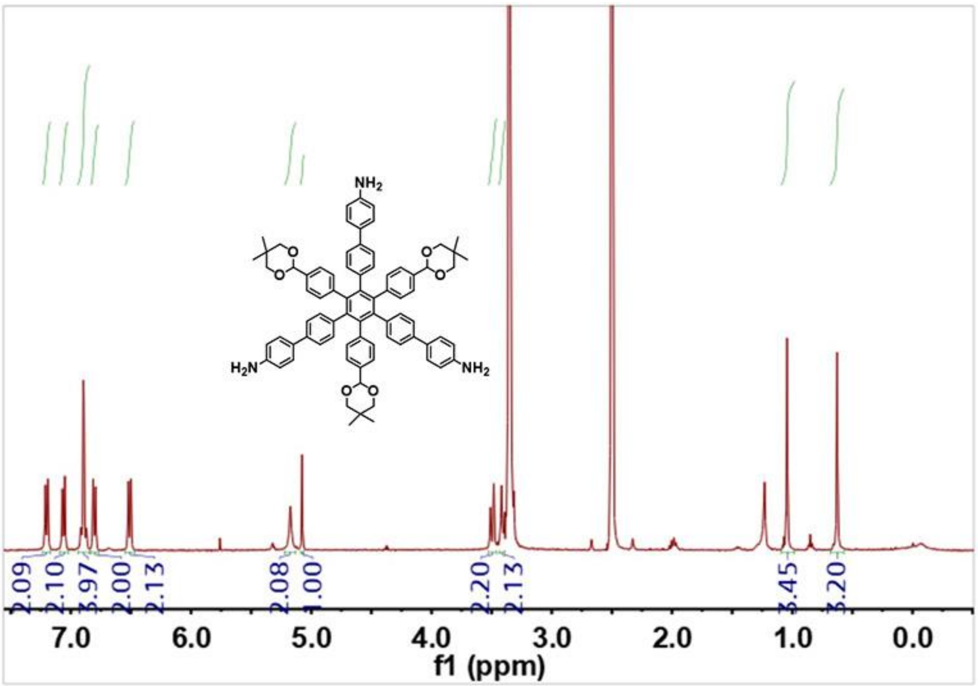

Figure S23. ^{1}H NMR ($CDCl_3$, 400 MHz) spectra of 1,3,5-HPB-NH_2.

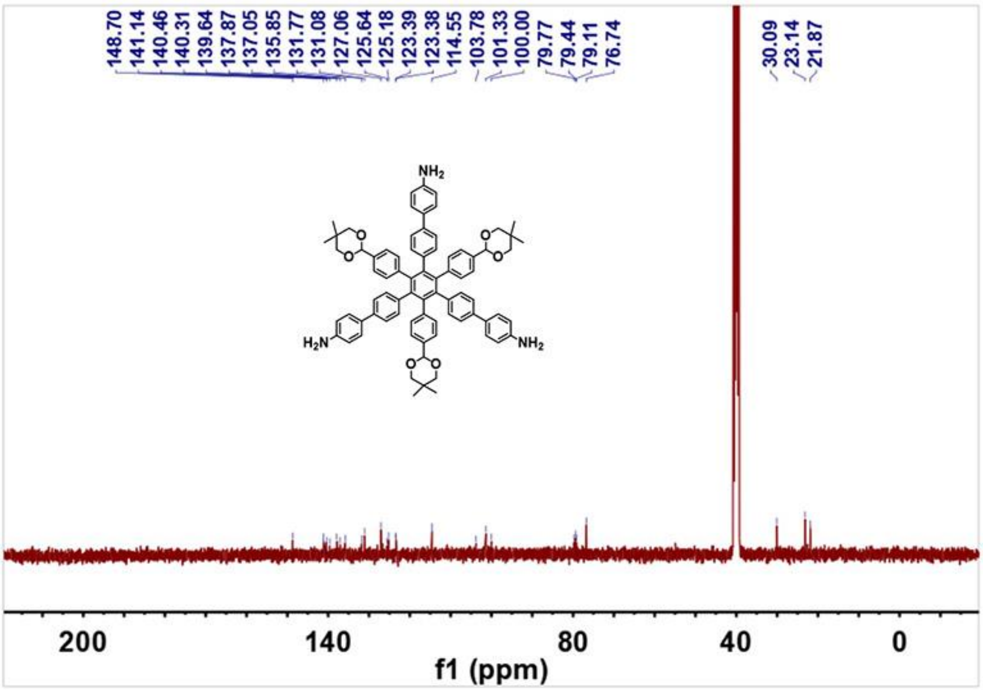

Figure S24. ^{13}C NMR ($CDCl_3$, 100 MHz) spectra of 1,3,5-HPB-NH_2.

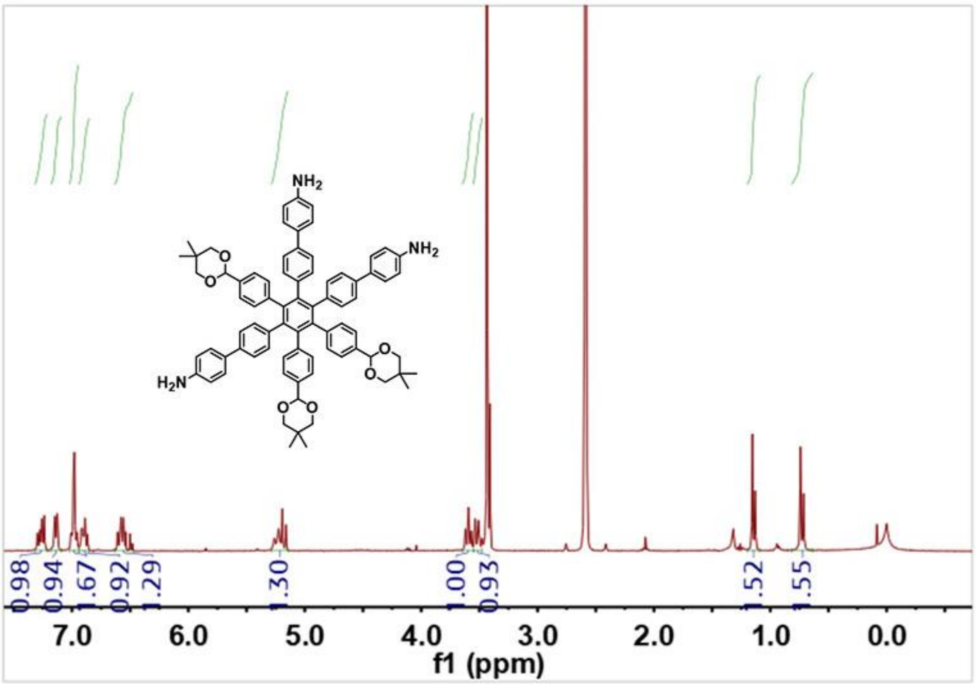

Figure S25. ^{1}H NMR ($CDCl_3$, 400 MHz) spectra of 1,2,4-HPB-NH_2.

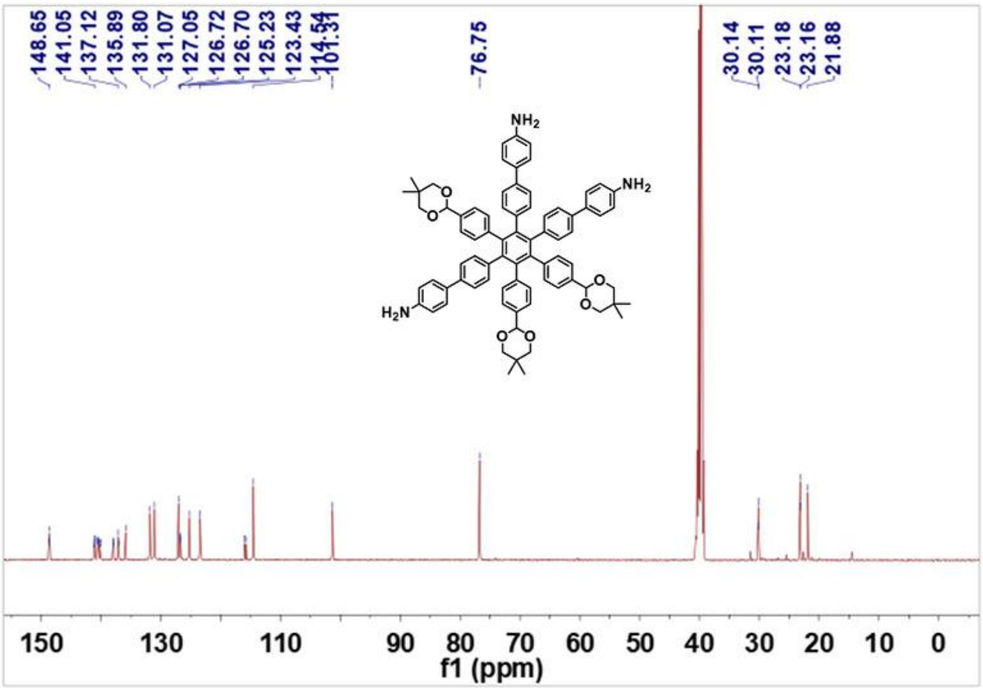

Figure S26. ^{13}C NMR ($CDCl_3$, 100 MHz) spectra of 1,2,4-HPB-NH_2.

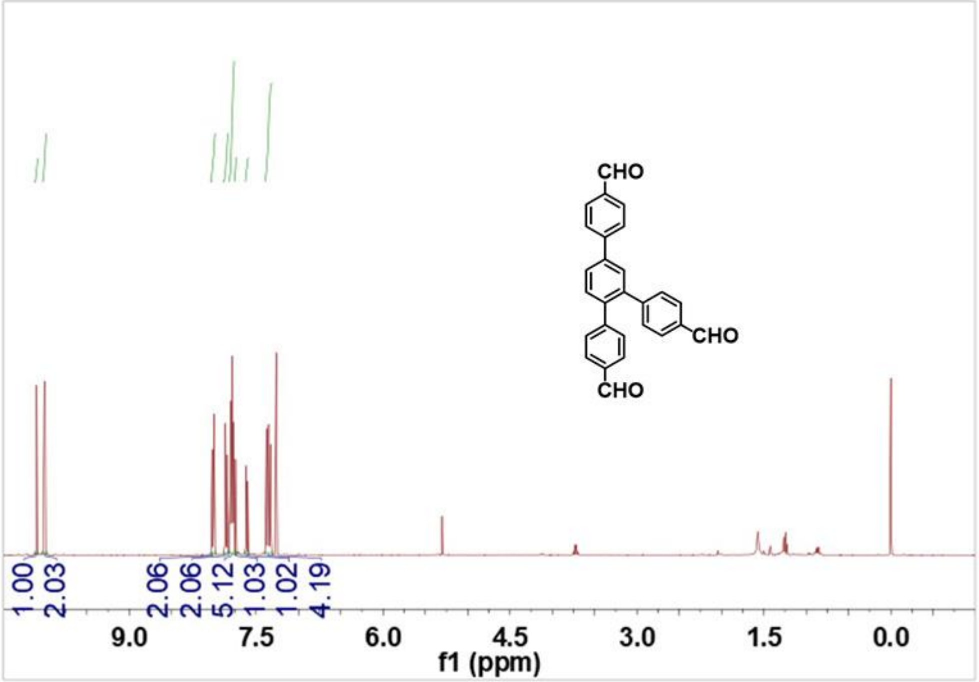

Figure S27. ^{1}H NMR ($CDCl_3$, 400 MHz) spectra of 1,2,4-TFB.

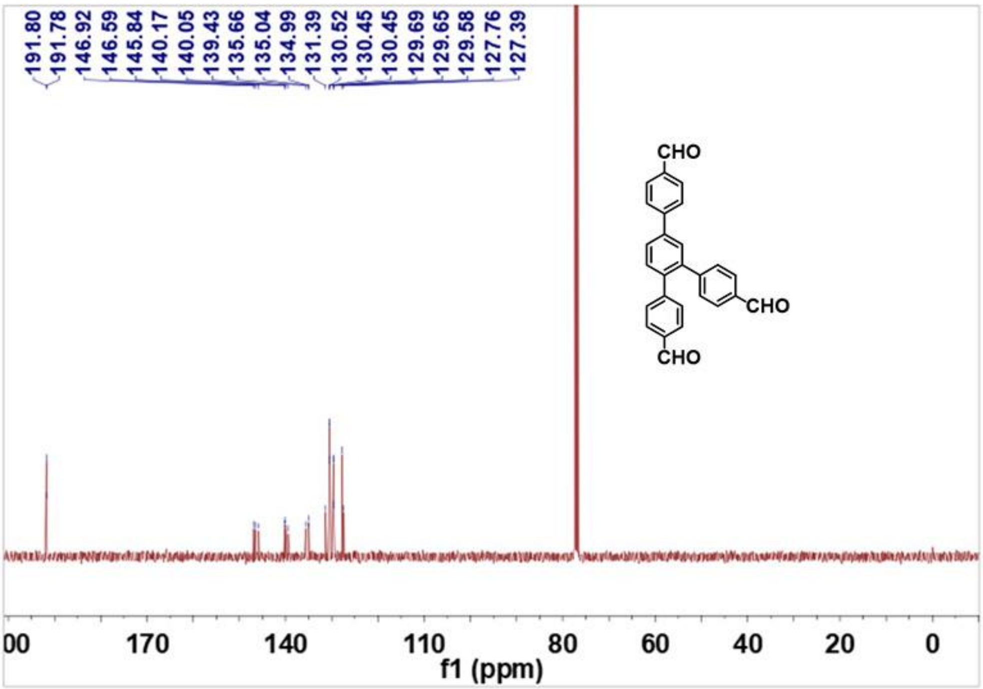

Figure S28. ^{13}C NMR ($CDCl_3$, 100 MHz) spectra of 1,2,4-TFB.

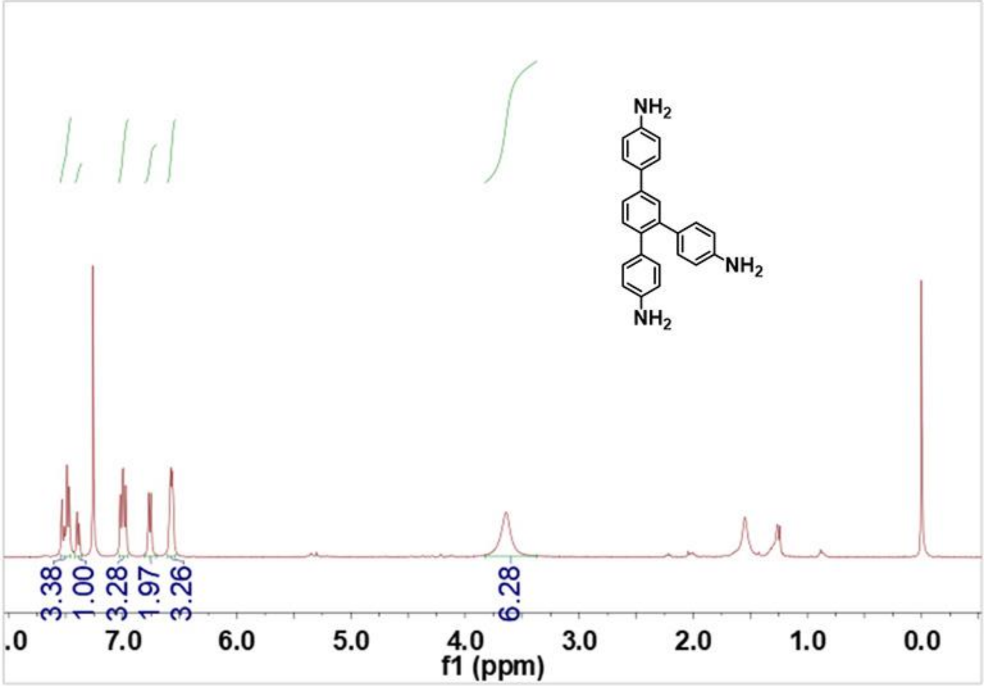

Figure S29. ^{1}H NMR ($CDCl_3$, 400 MHz) spectra of 1,2,4-TAB.

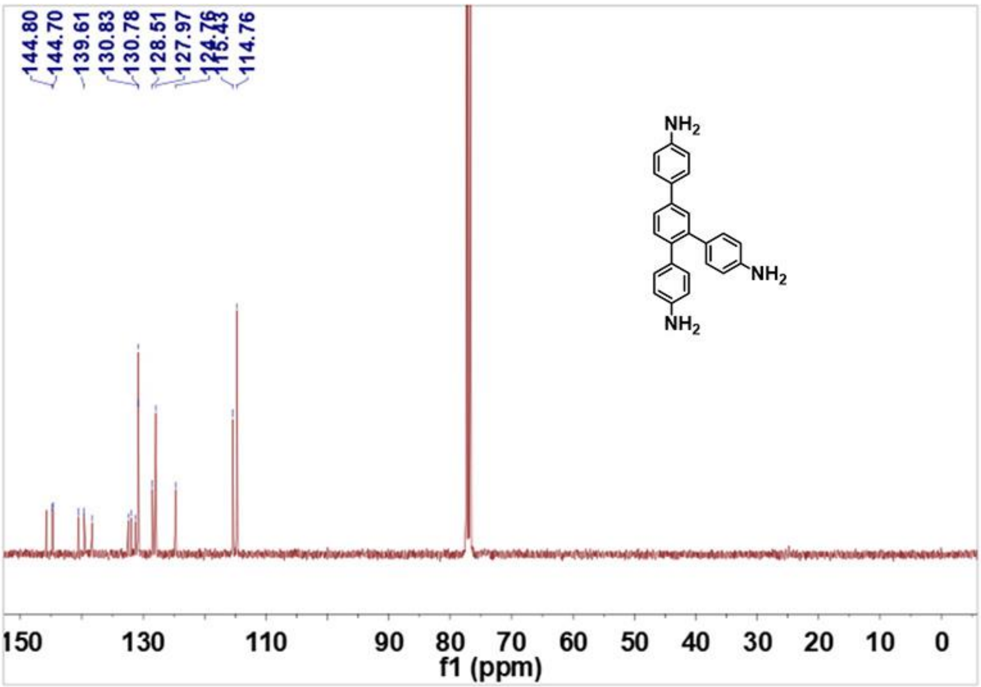

Figure S30. ^{13}C NMR ($CDCl_3$, 100 MHz) spectra of 1,2,4-TAB.

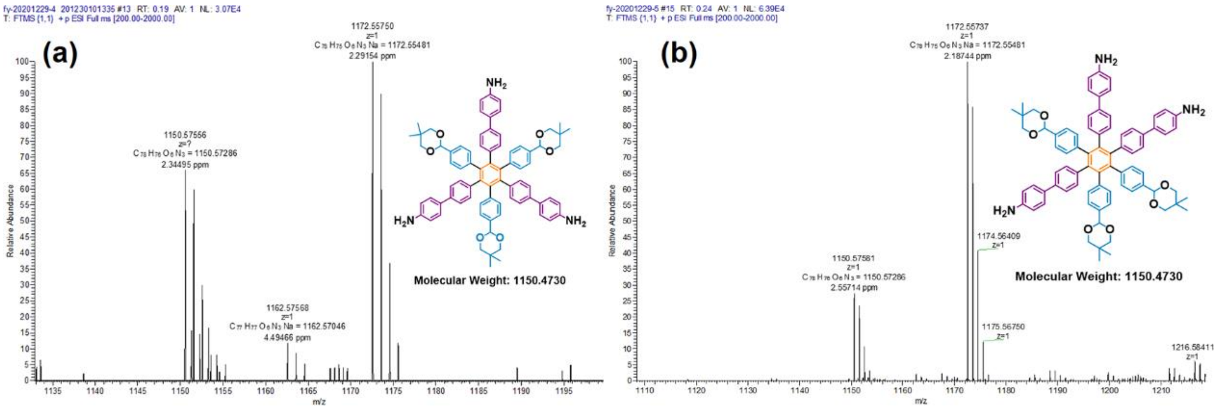

Figure S31. High-resolution mass spectra of (a) symmetric 1,3,5-HPB-NH_2 and (b) asymmetric 1,2,4-HPB-NH_2.

Table S2. Atomic coordinates of the simulated AA-stacking model of 1,3,5-HPB-COF

Space group: ***P1***<br>***a*** **= 19.4286 Å,** ***b*** **= 19.4372 Å, and** ***c*** **= 5.7109 Å.**<br>***α*** **= 90°,** ***β*** **= 90° and** ***γ*** **= 120°**			
	**X**	**Y**	**Z**
C	5.99745	-0.9277	-0.49541
C	5.99483	-0.85286	-0.51952
C	5.99171	-0.71067	-0.58129
C	5.96241	-0.76916	-0.75598
C	5.96464	-0.83918	-0.72562
C	5.82536	-0.97608	-0.30244
C	5.75192	-0.97946	-0.29868
C	5.60093	-0.98186	-0.67337
C	5.5294	-0.98105	-0.66729
C	5.07138	-0.9253	-0.49279
C	5.14685	-0.84768	-0.48555
C	5.15131	-0.99438	-0.51399
C	5.02426	-0.79463	-0.34368
C	5.02292	-0.72376	-0.3746
C	5.29598	-0.99255	-0.57641
C	5.26716	-0.96277	-0.75063
C	5.19527	-0.96437	-0.71984
C	5.19497	-0.8275	-0.28707
C	5.26648	-0.75578	-0.2753
C	5.29219	-0.70194	-0.46396
C	5.2428	-0.72217	-0.66326
C	5.1708	-0.79427	-0.67352
C	5.36984	-0.6256	-0.45136
C	5.37168	-0.99107	-0.61719
C	5.3823	-0.5579	-0.57755
C	5.45527	-0.48693	-0.56539
C	5.51636	-0.48152	-0.42217

C	5.50408	-0.54742	-0.29228
C	5.43247	-0.6193	-0.31037
C	5.92583	-0.0018	-0.49484
C	5.92819	-0.07338	-0.49249
C	5.84801	-0.0044	-0.49584
C	5.85295	-0.15093	-0.50762
C	5.69982	-0.01137	-0.48928
C	5.72249	-0.0396	-0.68397
C	5.79616	-0.03607	-0.68721
C	5.79697	-0.17699	-0.32574
C	5.72459	-0.24805	-0.34821
C	5.70723	-0.29362	-0.5535
C	5.76339	-0.26754	-0.73455
C	5.8357	-0.19683	-0.71125
C	6.03574	-0.16692	-0.68302
C	6.0385	-0.23744	-0.67999
C	5.63058	-0.36748	-0.58346
C	5.62268	-0.01363	-0.48607
C	5.97961	-0.41683	-0.67113
C	5.97886	-0.48914	-0.66688
C	5.47772	-0.01313	-0.47394
C	5.49951	-0.04523	-0.28828
C	5.57144	-0.04537	-0.29321
N	5.40385	-0.0131	-0.45577
C	5.98935	-0.63682	-0.62062
N	5.59088	-0.40916	-0.40312
C	5.00219	-0.07099	-0.49154
C	5.07375	0.00299	-0.49169
C	5.00459	-0.14628	-0.49136
C	5.00991	-0.28836	-0.48543
C	4.97858	-0.26767	-0.29434
C	4.97603	-0.19716	-0.29764
C	5.17974	-0.02425	-0.33833
C	5.2517	-0.02351	-0.36951
C	5.01143	-0.36365	-0.48299
C	5.01103	-0.50907	-0.47453
C	5.04299	-0.45562	-0.28776
C	5.04309	-0.38358	-0.29087
N	5.01147	-0.58276	-0.45868
H	5.93787	-0.76061	-0.9164
H	5.94351	-0.88294	-0.86322
H	5.86473	-0.95158	-0.15486
H	5.7362	-0.956	-0.14889
H	5.64042	-0.95575	-0.82161
H	5.5163	-0.95409	-0.81262
H	5.04898	-0.80381	-0.18482
H	5.04657	-0.67894	-0.23859
H	5.30019	-0.93849	-0.91131
H	5.17272	-0.94331	-0.85741
H	5.17643	-0.86759	-0.14077
H	5.3009	-0.74265	-0.11628

H	5.25996	-0.68311	-0.81319
H	5.13372	-0.80865	-0.82768
H	5.39928	-0.97106	-0.78662
H	5.3355	-0.55884	-0.68248
H	5.46375	-0.43549	-0.66266
H	5.55131	-0.54323	-0.18184
H	5.42713	-0.66993	-0.21583
H	5.68267	-0.06531	-0.83149
H	5.81273	-0.05848	-0.83743
H	5.80893	-0.14148	-0.16918
H	5.68129	-0.26686	-0.20822
H	5.75145	-0.3023	-0.8931
H	5.87806	-0.17681	-0.85292
H	6.05837	-0.12802	-0.83347
H	6.06389	-0.25185	-0.82768
H	5.60996	-0.38902	-0.75952
H	5.95357	-0.40313	-0.81889
H	5.95205	-0.52888	-0.81299
H	5.46019	-0.0701	-0.13933
H	5.58686	-0.07083	-0.14777
H	5.96869	-0.62937	-0.78915
H	4.95486	-0.30684	-0.14464
H	4.95172	-0.182	-0.14976
H	5.14568	-0.04897	-0.17979
H	5.2726	-0.04756	-0.2339
H	5.06795	-0.47031	-0.13955
H	5.06864	-0.34298	-0.14488

Table S3. Refined atomic coordinates of the simulated AA-stacking model of 1,3,5-HPB-COF.

Space group: P1<br>***a* = 19.296 Å, *b* = 19.293 Å, *c* = 5.661 Å**<br>***α* = 90.0009°, *β* = 89.9986°, *γ* = 120.7114°**			
	**X**	**Y**	**Z**
C	6.04186	-0.84117	-0.49603
C	6.03922	-0.76637	-0.52013
C	6.03608	-0.62421	-0.5819
C	6.00656	-0.68313	-0.75661
C	6.00881	-0.75312	-0.72625
C	5.8685	-0.89204	-0.30302
C	5.79451	-0.89648	-0.29925
C	5.6424	-0.90106	-0.67398
C	5.57034	-0.90128	-0.66789
C	5.10893	-0.85214	-0.49333
C	5.18496	-0.77343	-0.48609
C	5.18945	-0.92007	-0.51454
C	5.06146	-0.72214	-0.34419
C	5.06011	-0.65129	-0.37511

C	5.3352	-0.91615	-0.57698
C	5.30616	-0.88679	-0.75122
C	5.23374	-0.88943	-0.72042
C	5.23344	-0.75255	-0.28759
C	5.30548	-0.6798	-0.27582
C	5.33138	-0.62558	-0.4645
C	5.28162	-0.64653	-0.66382
C	5.20909	-0.71967	-0.67408
C	5.4096	-0.54812	-0.4519
C	5.41146	-0.91358	-0.61777
C	5.42215	-0.48023	-0.5781
C	5.49567	-0.4082	-0.56594
C	5.55721	-0.40191	-0.42271
C	5.54484	-0.46799	-0.29281
C	5.4727	-0.54091	-0.3109
C	5.96971	0.08375	-0.4954
C	5.97209	0.0122	-0.49305
C	5.89131	0.08002	-0.49639
C	5.89629	-0.06644	-0.50818
C	5.74203	0.07091	-0.48982
C	5.76486	0.04301	-0.68454
C	5.83908	0.0476	-0.68778
C	5.8399	-0.09331	-0.32628
C	5.76698	-0.16542	-0.34875
C	5.74949	-0.21125	-0.55406
C	5.80607	-0.18435	-0.73514
C	5.87891	-0.1126	-0.71184
C	6.08043	-0.0798	-0.68362
C	6.08321	-0.15028	-0.68059
C	5.67227	-0.28622	-0.58402
C	5.66431	0.06754	-0.4866
C	6.02389	-0.33053	-0.67174
C	6.02313	-0.40286	-0.66749
C	5.51828	0.06595	-0.47446
C	5.54023	0.03416	-0.28878
C	5.6127	0.03506	-0.29372
N	5.44386	0.06491	-0.45628
C	6.0337	-0.55039	-0.62123
N	5.63228	-0.32847	-0.40366
C	5.03923	0.00122	-0.49202
C	5.11132	0.07624	-0.49218
C	5.04165	-0.07404	-0.49185
C	5.04701	-0.21605	-0.48593
C	5.01545	-0.19581	-0.29481
C	5.01288	-0.12534	-0.29811
C	5.21809	0.05052	-0.33881
C	5.29059	0.0523	-0.37
C	5.04854	-0.29132	-0.48349
C	5.04814	-0.43676	-0.47504
C	5.08033	-0.38284	-0.28824

C	5.08043	-0.3108	-0.29135
N	5.04858	-0.51045	-0.45919
H	5.98032	-0.67392	-0.92601
H	5.98551	-0.80069	-0.87155
H	5.91125	-0.86524	-0.14628
H	5.77641	-0.87334	-0.1394
H	5.68403	-0.87501	-0.83336
H	5.55322	-0.87464	-0.82116
H	5.08056	-0.74116	-0.17744
H	5.08141	-0.61228	-0.20938
H	5.34208	-0.86035	-0.92066
H	5.20975	-0.86623	-0.86537
H	5.2142	-0.79555	-0.13077
H	5.34455	-0.66346	-0.11061
H	5.30037	-0.60343	-0.82029
H	5.16939	-0.73541	-0.83772
H	5.44343	-0.89224	-0.7952
H	5.37183	-0.48364	-0.69229
H	5.50576	-0.3539	-0.67372
H	5.59406	-0.4628	-0.16984
H	5.46418	-0.59593	-0.21036
H	5.72255	0.01625	-0.84225
H	5.85746	0.02475	-0.84772
H	5.85342	-0.05613	-0.15794
H	5.72115	-0.18733	-0.1988
H	5.79267	-0.22135	-0.90381
H	5.92517	-0.091	-0.86058
H	6.10891	-0.03543	-0.83611
H	6.11414	-0.16371	-0.83032
H	5.64678	-0.30791	-0.76867
H	6.00324	-0.31221	-0.83668
H	6.001	-0.44436	-0.82797
H	5.49863	0.0069	-0.12981
H	5.63015	0.00912	-0.13936
H	6.01785	-0.53717	-0.8032
H	4.99155	-0.23729	-0.13451
H	4.987	-0.10906	-0.14097
H	5.18219	0.0249	-0.1689
H	5.31377	0.02792	-0.22613
H	5.10711	-0.39745	-0.12981
H	5.10671	-0.26728	-0.13611
